# Estimated global and regional causes of deaths from diarrhoea in children younger than 5 years during 2000–21: a systematic review and Bayesian multinomial analysis

**DOI:** 10.1016/S2214-109X(24)00078-0

**Published:** 2024-04-20

**Authors:** Robert E Black, Jamie Perin, Diana Yeung, Tushara Rajeev, Jacob Miller, Sarah E Elwood, James A Platts-Mills

**Affiliations:** a Department of International Health, Bloomberg School of Public Health, Johns Hopkins University, Baltimore, MD, USA; b Division of Infectious Diseases and International Health, University of Virginia, Charlottesville, VA, USA

## Abstract

**Background:**

Information on the causes of deaths from diarrhoea in children younger than 5 years is needed to design improved preventive and therapeutic approaches. We aimed to conduct a systematic analysis of studies to report estimates of the causes of deaths from diarrhoea in children younger than 5 years at global and regional levels during 2000–21.

**Methods:**

For this systematic review and Bayesian multinomial analysis, we included 12 pathogens with the highest attributable incidence in the Global Enteric Multicenter Study. We searched PubMed, Scopus, Embase, Web of Science, Global Health Index Medicus, Global Health OVID, IndMed, Health Information Platform for the Americas (PLISA), Africa-Wide Information, and Cochrane Collaboration for articles published between Jan 1, 2000, and Dec 31, 2020, using the search terms “child”, “hospital”, “diarrhea”, “diarrhoea”, “dysentery”, “rotavirus”, “*Escherichia coli*”, “salmonella”, “shigella”, “campylobacter”, “*Vibrio cholerae*”, “cryptosporidium”, “norovirus”, “astrovirus”, “sapovirus”, and “adenovirus”. To be included, studies had to have a patient population of children younger than 5 years who were hospitalised for diarrhoea (at least 90% of study participants), at least a 12-month duration, reported prevalence in diarrhoeal stools of at least two of the 12 pathogens, all patients with diarrhoea being included at the study site or a systematic sample, at least 100 patients with diarrhoea, laboratory tests done on rectal swabs or stool samples, and standard laboratory methods (ie, quantitative PCR [qPCR] or non-qPCR). Studies published in any language were included. Studies were excluded if they were limited to nosocomial, chronic, antibiotic-associated, or outbreak diarrhoea or to a specific population (eg, only children with HIV or AIDS). Each article was independently reviewed by two researchers; a third arbitrated in case of disagreement. If both reviewers identified an exclusion criterion, the study was excluded. Data sought were summary estimates. Data on causes from published studies were adjusted when necessary to account for the poor sensitivity of non-qPCR methods and for attributable fraction based on quantification of pathogens in children who are ill or non-ill. The causes of deaths from diarrhoea were modelled on the causes of hospitalisations for diarrhoea. We separately modelled studies reporting causes of diarrhoea in children who were hospitalised in low-income and middle-income countries (LMICs) and in high-income countries (HICs).

**Findings:**

Of 74 282 papers identified in the initial database search, we included 138 studies (91 included data from LMICs and 47 included data from HICs) from 73 countries. We modelled estimates for 194 WHO member states (hereafter referred to as countries), including 42 HICs and 152 LMICs. We could attribute a cause to 1 003 448 (83·8%) of the estimated 1 197 044 global deaths from diarrhoea in children younger than 5 years in 2000 and 360 730 (81·3%) of the estimated 443 833 global deaths from diarrhoea in children younger than 5 years in 2021. The cause with the largest estimated global attribution was rotavirus; in LMICs, the proportion of deaths from diarrhoea due to rotavirus in children younger than 5 years appeared lower in 2021 (108 322 [24·4%] of 443 342, 95% uncertainty interval 21·6–29·5) than in 2000 (316 382 [26·5%] of 1 196 134, 25·7–28·5), but the 95% CIs overlapped. In 2000, the second largest estimated attribution was norovirus GII (95 817 [8·0%] of 1 196 134 in LMICs and 225 [24·7%] of 910 in HICs); in 2021, *Shigella* sp had the second largest estimated attribution in LMICs (36 082 [8·1%] of 443 342), but norovirus remained with the second largest estimated attribution in HICs (84 [17·1%] of 490).

**Interpretation:**

Our results indicate progress in the reduction of deaths from diarrhoea caused by 12 pathogens in children younger than 5 years in the past two decades. There is a need to increase efforts for prevention, including with rotavirus vaccine, and treatment to eliminate further deaths.

**Funding:**

Bill & Melinda Gates Foundation via Johns Hopkins University and the University of Virginia.

**Research in context**

**Evidence before this study**

We did a systematic search for articles on the prevalence of 12 pathogens associated with diarrhoea published between Jan 1, 2000, and Dec 31, 2020. We searched PubMed, Scopus, Embase, Web of Science, Global Index Medicus, Global Health OVID, IndMed, Health Information Platform for the Americas (PLISA), Africa-Wide Information, and Cochrane Collaboration using the search terms “child”, “hospital”, “diarrhea”, “diarrhoea”, “dysentery”, “rotavirus”, “*Escherichia coli*”, “salmonella”, “shigella”, “campylobacter”, “*Vibrio cholerae*”, “cryptosporidium”, “norovirus”, “astrovirus”, “sapovirus”, and “adenovirus”. Studies were included if they were of children younger than 5 years hospitalised for diarrhoea, included 100 children or more, had a duration of 12 or more consecutive months, included all patients at the study site or a systematic sample, and used conventional or quantitative PCR (qPCR) methods to test rectal swabs or stools for multiple pathogens. Studies published in any language were included. The definition of hospitalised was that used in the articles, but was generally considered to require an overnight stay. Studies were excluded if they were limited to nosocomial, chronic, antibiotic-associated, or outbreak diarrhoea or if they were limited to special populations, such as children with HIV or AIDS. There were two previous estimates of the global prevalence of pathogens associated with deaths from diarrhoea in children younger than 5 years. One was based on a review of the literature on diarrhoea of all severities; it included nine causes and 204 countries and territories. It found that, in 2019, the five most common causes were rotavirus, shigella, adenovirus, cryptosporidium, and *Vibrio cholerae*. These estimates did not include norovirus, astrovirus, or sapovirus. The other was based on a review of the literature on diarrhoea in children admitted to hospital; it included 11 causes and 194 countries. It found that, in 2011, the five most common causes were rotavirus, typical enteropathogenic *Escherichia coli*, norovirus, enterotoxigenic *E coli*, and shigella. These estimates did not include sapovirus.

**Added value of this study**

Our study used a comprehensive review of the literature to establish the prevalence of pathogens in children hospitalised for diarrhoea. The study made important adjustments to these data to account for advances in laboratory methods for detection and quantification of pathogens in children who are ill or non-ill. The use of highly sensitive qPCR methods for the detection of pathogens provided a basis for the use of odds ratios comparing the presence of pathogens in children with diarrhoea versus children who were asymptomatic to estimate the proportion of instances of diarrhoea or deaths that could be attributed to each pathogen. The two previous estimates used published results with a range of diagnostic methods, including conventional microbiology, without appropriately accounting for the increased sensitivity of more recently applied qPCR methods or for the identification of pathogens in children who were non-ill.

**Implications of all the available evidence**

Despite substantial declines in childhood deaths from diarrhoea since 2000, more than 400 000 deaths from diarrhoea caused by 12 pathogens occurred in 2021, more than 99% of which were in low-income and middle-income countries. The high prevalence of rotavirus as a cause of death indicates the need to increase the coverage of existing vaccines while developing vaccines with increased efficacy in high-mortality settings. The use of cholera vaccines might be justified in some settings to prevent and interrupt epidemics. Vaccines against the other 10 pathogens would prevent only a small proportion of global deaths from diarrhoea in children younger than 5 years, making investment in vaccine development difficult to justify. Policy and intervention-programme priority should be given to general preventive measures, such as water availability and quality, sanitation, personal hygiene, food safety, and access to effective treatment.

## Introduction

Deaths from diarrhoea in children younger than 5 years declined by 83% from an estimated 2·7 million in 1980 to an estimated 443 833 in 2021,^1^, ^2^ partly because of improved treatment with oral-rehydration therapy and zinc.^3^ Prevention of diarrhoea would not only reduce the number of deaths, but also morbidity and adverse effects on nutrition and development. The only vaccines against diarrhoea that are recommended for routine use are for the prevention of rotavirus.^4^ Information on the causes of deaths from diarrhoea is crucial to understand the need for and design of additional vaccines and specific therapies.

Numerous agents that cause diarrhoea have been historically identified via bacterial culture, antigen detection, microscopy, nucleic-acid amplification, and immunological methods.^5^ Quantitative PCR (qPCR) is a highly sensitive and reproducible method that has become the preferred approach for studies of the infectious causes of diarrhoea. Use of qPCR in such studies has frequently identified one or more possible causal agents of moderate-to-severe diarrhoea or diarrhoea that requires admission to hospital in children younger than 5 years, but also in non-ill, matched-control individuals, necessitating the use of attributable risk to assign causes with specificity.^6^, ^7^

The availability of evidence from the past 10 years allows us to make adjustments to results found in the literature, thereby allowing improved estimates of causes.

We aimed to conduct a systematic review and Bayesian multinomial analysis of studies that reported the prevalence of diarrhoeagenic pathogens in children hospitalised for diarrhoea, assuming that they would have a similar causal distribution to fatal diarrhoea. To do so accurately, we aimed to extract the results and diagnostic approaches of individual studies and adjust for both the poor sensitivity of non-PCR diagnostics and possible subclinical detection in these settings. We ultimately aimed to report estimates of the causes of deaths from diarrhoea in children younger than 5 years at global and regional levels during 2000–21.

## Methods

### Search strategy and selection criteria

For this systematic review and Bayesian multinomial analysis, we included 12 pathogens with the highest attributable incidence in the Global Enteric Multicenter Study (GEMS; ie, adenovirus 40/41; astrovirus; *Campylobacter jejuni* or *Campylobacter coli*; *Cryptosporidium* sp; norovirus GII; rotavirus; *Salmonella* sp; sapovirus; *Shigella* sp; heat-stable, toxin-producing, enterotoxigenic *Escherichia coli* [ST-ETEC]; typical, enteropathogenic *E coli* [EPEC]; and *Vibrio cholerae*).^6^ We searched PubMed, Scopus, Embase, Web of Science, Global Health Index Medicus, Global Health OVID, IndMed, Health Information Platform for the Americas (PLISA), Africa-Wide Information, and Cochrane Collaboration for articles published between Jan 1, 2000, and Dec 31, 2020, using search terms related to these specific causes of diarrhoea (ie, “child”, “hospital”, “diarrhea”, “diarrhoea”, “dysentery”, “rotavirus”, “*Escherichia coli*”, “salmonella”, “shigella”, “campylobacter”, “*Vibrio cholerae*”, “cryptosporidium”, “norovirus”, “astrovirus”, “sapovirus”, and “adenovirus”; appendix 1 p 2). To be included, studies had to have a patient population of children younger than 5 years who were hospitalised for diarrhoea (at least 90% of study participants), at least a 12-month duration, reported prevalence in diarrhoeal stools of at least two of the 12 pathogens, all patients with diarrhoea being included at the study site or a systematic sample, at least 100 patients with diarrhoea, laboratory tests done on rectal swabs or stool samples, and standard laboratory methods (ie, qPCR or non-qPCR [hereafter referred to as conventional]). Studies published in any language were included; translation was done via people who spoke the language or Google Translate. The definition of hospitalised was that used in the articles, but was generally considered to require an overnight stay. Studies were excluded if they were limited to nosocomial, chronic, antibiotic-associated, or outbreak diarrhoea or to a specific population (eg, only children with HIV or AIDS). We only included peer-reviewed papers, and extracted summary estimates.

No ethics approval was needed as we used entirely published results.

We used DistillerSR systematic-review artificial-intelligence (AI) software (DistillerSR, Ottawa, ON, Canada)^8^ during title and abstract screening to reorder references on the basis of relevance.^9^ References were excluded by the AI if the likelihood of inclusion was less than 5%. Each article was independently reviewed by two graduate-level researchers (TR and JM) and a third (DY) arbitrated in case of disagreement. The reviewers identified exclusion criteria, looking first for inclusion of at least two pathogens and whether the children were admitted to hospital, and recorded up to three reasons for exclusion per study. Studies could have had more reasons for exclusion that were not recorded. Duplicate data were excluded. Of the PRISMA Checklist 2020, not all items were applicable to our search because our highly selective inclusion and exclusion criteria were designed to address possible bias and because we used the results of the review as input to a Bayesian model rather than a meta-analysis (appendix 1 pp 3–5). The prevalence of each pathogen, including co-infections, was extracted from each reference.

### Data analysis

Re-analyses of two large, multisite studies of the causes of diarrhoea in children younger than 5 years in low-resource settings (ie, GEMS and Etiology, Risk Factors, and Interactions of Enteric Infections and Malnutrition and the Consequences for Child Health [MAL-ED]) with qPCR diagnostics led to important changes in their estimates.^6^, ^7^ Both studies tested stool samples by qPCR and conventional diagnostic methods and tested diarrhoeal and non-diarrhoeal stools, and thus could incorporate the detection of pathogens in the absence of diarrhoea by calculating a cause-specific attributable fraction via the association between pathogen quantity and diarrhoea. For our study, we needed adjustments to estimate the attribution of cause on the basis of literature-derived prevalence data. Therefore, we used participant-level data from GEMS and MAL-ED^6^, ^7^ to adjust for detection of each pathogen with conventional versus qPCR diagnostics and to adjust for expected detection of pathogens in the absence of diarrhoea.

For each pathogen, we selected either the GEMS or MAL-ED datasets to adjust for comparative detection by conventional and qPCR diagnostics. We used GEMS preferentially because it was facility-based and the diagnostics were similar to those in studies of children who were hospitalised. We used MAL-ED for the adjustment only if the conventional diagnostic was more representative of the diagnostics used in studies found in the literature than those used in GEMS (appendix 1 p 7). The results of MAL-ED were not included for prevalence of pathogens as it was community-based.

We used GEMS and MAL-ED data to model the association between pathogen quantity and diarrhoea and calculate attributable fractions. Model development and model application to other studies have been described previously.^6^, ^7^, ^10^ Briefly, by use of a conditional logistic-regression model for GEMS and a generalised linear mixed-effects model with a random effect for each individual for MAL-ED, we fit a new model for each pathogen to describe the association between pathogen quantity and diarrhoea with a random slope for site to allow for variation in the strength of association between pathogen quantity and diarrhoea. The MAL-ED model was additionally adjusted for sex, age, and TaqMan Array (Thermo Fisher Scientific, Waltham, MA, USA) card batch. These adjustments were not needed for GEMS as control individuals were already matched on age and sex with children with diarrhoea. Pathogen quantity was defined as the log10 increase in pathogen quantity above the analytical cutoff based on the quantification cycle (*C*_q_), namely

$$\frac{35-Cq}{\log2\left(10\right)}$$

A weighted population-attributable fraction for each pathogen was then calculated for any stratum of *j* as

$$\text{AF}=\text{1}-=\frac{\sum_{\text{1}}^{\text{j}}\frac{wt_{i}}{OR_{i}}}{\sum_{1}^{j}wt_{i}}$$

where *wt*_i_ was the episode-specific inverse probability weight and *OR*_i_ was the episode-specific and quantity-specific odds ratio (OR) derived from the regression model.^11^ We propagated uncertainty from the attributable-fraction estimates using a Monte Carlo approach,^10^ which simulated 1000 new OR estimates from a normal distribution with the mean derived from the model coefficients and variance-covariance from the covariance matrix.

GEMS and MAL-ED were both conducted in areas of high endemicity for enteric pathogens. To optimise the alignment between the study settings for literature-derived prevalence estimates and the GEMS and MAL-ED sites, we weighted the 1000 simulations towards the GEMS and MAL-ED sites that were most representative of the expected pathogen density in studies from the literature. We used pathogen prevalence data from the Global Pediatric Diarrhoea Surveillance (GPDS) network, which is broadly representative of low-income and middle-income countries (LMICs).^10^ Specifically, we calculated a draw distribution for each GPDS site by deriving weights that minimised the root mean-square error distance between the pathogen density distribution in children with diarrhoea from that site and the proportionally weighted aggregate distribution across the GEMS and MAL-ED sites. We classified GPDS sites by World Bank income category (ie, low income, lower-middle income, and upper-middle income)^12^ and draw distributions for each category were derived from the mean weight for each pathogen across all sites in each category.

Scalars were then calculated for the adjustment from qPCR detection to qPCR attributable fraction as

$$Scalar_{qPCR}=\frac{{\text{AF}}_{qPCR}}{Prevalence_{qPCR}}$$

and for adjustment from conventional detection to qPCR attributable fraction as

$$Scalar_{conventional}=\frac{{\text{AF}}_{qPCR}}{Prevalence_{conventional}}$$

For each pathogen and income category, 1000 estimates of each scalar type were derived, one from each model simulation. The mean was calculated for each diagnostic method and for the qPCR scalar by country income category. For each pathogen, we used the conventional scalar to estimate attributable fractions for studies in which prevalence with a conventional method was reported; we used the qPCR scalar for studies where the prevalence by qPCR was reported. If a study included healthy control individuals and presented attributable fractions, these were used directly without adjustment in the analysis (appendix 1 pp 8–9).

The causes of deaths from diarrhoea were modelled on the causes of hospitalisations for diarrhoea. The distribution of causes was assumed to be multinomial and was modelled in a Bayesian framework, in which some causes (or multinomial categories) were not reported as many studies reported fewer than the 12 causes of interest.^13^ We included 12 pathogens and a residual category (hereafter referred to as other). The multinomial model constrained the sum of the causal fractions to 1.

We separately modelled studies reporting causes of diarrhoea in children who were hospitalised in LMICs and in high-income countries (HICs). We used the total number of children hospitalised with diarrhoea reported, except for extremely large studies, in which we used the causal fractions but limited the number to 1000 children in the model so that we did not overweight specific studies. For the model of LMIC study data, we included gross national per-capita income (GNI),^14^ WHO region where the study was conducted (excluding HICs in all regions), rotavirus-vaccine coverage, and age of the children included in the study as covariates. For the age adjustment, we included indicators for the lower age limit being less than 2 years and for the upper age limit being more than 2 years and less than 5 years, with the vaccination at the midpoint for multiyear studies. Rotavirus vaccination coverage at the national level was used, with the most recent estimates being extrapolated with a flat trend up to 2021 and linearly interpolated for gaps for particular years within a country.^15^, ^16^ We also examined year and mortality rate in children younger than 5 years; however, these were highly collinear with other factors and we preferred to keep the number of covariates low because of the small number of studies. For parameters other than intercepts (pertaining to each cause), the prior distribution, including a shrinkage parameter for the amount of parameter restriction in the Least Absolute Shrinkage and Selection Operator (LASSO) regression,^13^ was chosen for a moderate amount of restriction (λ at 75) to prevent overfitting (appendix 1 pp 11–12). For the number of deaths from diarrhoea during 2000–21, we used our previously published estimates as the included studies did not report population-level rates of death.^1^, ^2^ These estimates included HICs.

For HICs, we fit a model in the same Bayesian framework. This model also incorporated the LASSO to prevent overfitting with a similar degree of restriction (λ at 150) with covariates for age and study year, while excluding the covariates for region, GNI, and coverage of rotavirus vaccine due to insufficient information on vaccine coverage. We did not include pathogens reported in fewer than ten studies in HICs; therefore typical EPEC, ST-ETEC, *Cryptosporidium* sp, and sapovirus were not estimated for HICs.

We used the mean of the posterior distribution for parameters from the Bayesian models of studies in HICs and in LMICs to estimate the causal distribution of deaths from diarrhoea for countries for years 2000 to 2021. We applied this causal distribution to the estimated number of deaths from diarrhoea in each country during 2000–21 to estimate the number of deaths for each cause.^1^

We established uncertainty by resampling from the draws of the posterior distribution of model parameters and estimating analogous draws of causes for each country in 2000 and 2021 as well as resampling from the posterior distribution of the number of country-specific deaths from diarrhoea in 2000 and 2021 and applying the causal percentage to the number of deaths from diarrhoea. These country-specific causal deaths were aggregated by WHO region and by national income group (ie, LMICs *vs* HICs). The uncertainty intervals were estimated by taking the 2·5th and 97·5th percentiles from the distribution of the number of deaths by cause and for the number of deaths from diarrhoea overall.

We did not assess risk of bias. Instead, we used highly restrictive inclusion and exclusion criteria that were designed to reduce bias.

All statistical analyses were conducted in R version 4.2.0 (appendix 1 pp 13–21).

### Role of the funding source

The funder of the study had no role in the study design, data collection, data analysis, data interpretation, or writing of the report.

## Results

**Figure 1:**
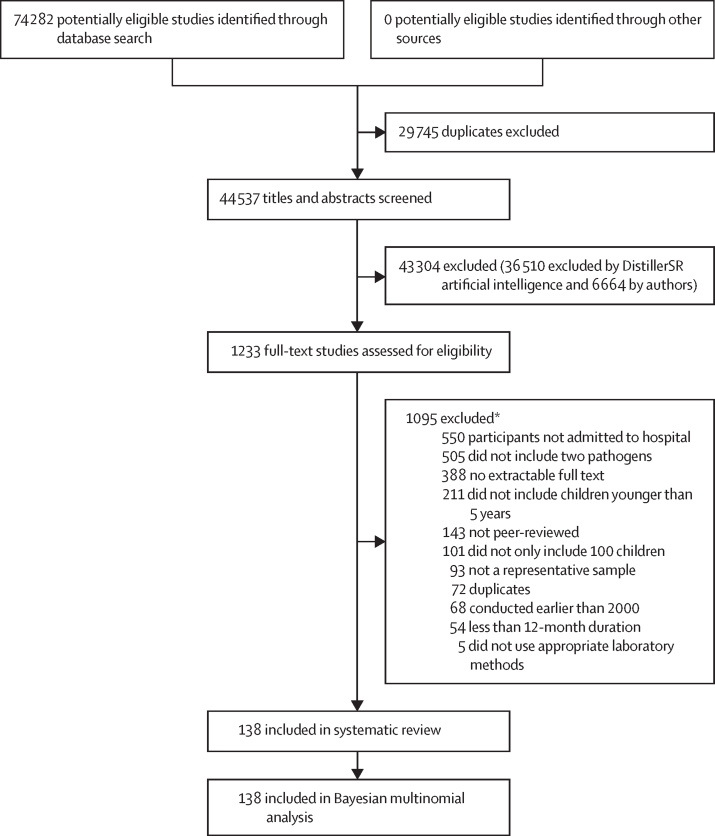
Study selection *Studies could be excluded for up to three reasons.

Of 74 282 papers identified in the initial database search, we were able to include 138 studies (91 included data from LMICs and 47 included data from HICs) from 73 countries in both the systematic review and Bayesian multinomial analysis that modelled estimates of the causes of death from diarrhoea for 194 WHO member states (hereafter referred to as countries), including 42 HICs and 152 LMICs (figure 1; appendix 1 pp 6–7). These studies included data during 2017–18 from 33 sites (28 countries) of the GPDS network.^10^

We could attribute a cause to 1 003 448 (83·8%) of the estimated 1 197 044 global deaths from diarrhoea in children younger than 5 years in 2000 and 360 730 (81·3%) of the estimated 443 833 global deaths from diarrhoea in children younger than 5 years in 2021 (table 1). The proportion not attributed to a cause was referred to as other and was presumed to represent causes that were not estimated separately, causes that were not detected because of insensitive laboratory methods, causes that are not currently shown to cause diarrhoea, or non-infectious causes.

**Table 1:** Estimated number of deaths and proportion of deaths from diarrhoea in children younger than 5 years by cause globally in 2000 and 2021

	**Global**		**High-income countries**		**Low-income and middle-income countries**	
	Estimated number of deaths, thousands	Proportion of deaths from diarrhoea	Estimated number of deaths, thousands	Proportion of deaths from diarrhoea	Estimated number of deaths, thousands	Proportion of deaths from diarrhoea
**2000**						
Rotavirus	316·634 (307·845–341·341)	26·5% (25·7–28·5)	0·252 (0·230–0·270)	27·6% (25·3–29·7)	316·382 (307·591–341·091)	26·5% (25·7–28·5)
Norovirus GII	96·043 (90·247–107·854)	8·0% (7·5–9·0)	0·225 (0·196–0·261)	24·7% (21·5–28·6)	95·817 (90·029–107·618)	8·0% (7·5–9·0)
Astrovirus	84·676 (77·460–95·532)	7·1% (6·5–8·0)	0·034 (0·030–0·038)	3·7% (3·3–4·2)	84·643 (77·424–95·496)	7·1% (6·5–8·0)
*Shigella* sp	78·781 (74·549–86·945)	6·6% (6·2–7·3)	0·028 (0·023–0·032)	3·0% (2·6–3·5)	78·753 (74·521–86·916)	6·6% (6·2–7·3)
Adenovirus 40/41	77·803 (71·006–89·211)	6·5% (5·9–7·5)	0·067 (0·058–0·075)	7·4% (6·4–8·3)	77·736 (70·944–89·145)	6·5% (5·9–7·5)
Sapovirus*	72·573 (66·158–80·863)	6·1% (5·5–6·8)	..	..	72·573 (66·158–80·863)	6·1% (5·5–6·8)
*Campylobacter jejuni* or *Campylobacter coli*	65·089 (60·621–71·115)	5·4% (5·1–5·9)	0·047 (0·041–0·053)	5·2% (4·5–5·9)	65·042 (60·574–71·069)	5·4% (5·1–5·9)
ST-ETEC*	62·599 (54·238–73·696)	5·2% (4·5–6·2)	..	..	62·599 (54·238–73·696)	5·2% (4·5–6·2)
*Cryptosporidium* sp*	60·113 (54·141–68·781)	5·0% (4·5–5·7)	..	..	60·113 (54·141–68·781)	5·0% (4·5–5·8)
Typical EPEC*	38·056 (32·605–45·172)	3·2% (2·7–3·8)	..	..	38·056 (32·605–45·172)	3·2% (2·7–3·8)
*Vibrio cholerae*	30·205 (28·170–35·858)	2·5% (2·4–3·0)	0·000 (0·000–0·000)	0·0% (0·0–0·0)	30·205 (28·170–35·858)	2·5% (2·4–3·0)
*Salmonella* sp	20·876 (18·944–24·108)	1·7% (1·6–2·0)	0·044 (0·039–0·049)	4·8% (4·3–5·4)	20·832 (18·899–24·067)	1·7% (1·6–2·0)
Other	193·596 (188·691–220·603)	16·2% (15·8–18·4)	0·214 (0·194–0·239)	23·5% (21·3–26·3)	193·382 (188·483–220·390)	16·2% (15·8–18·4)
Total	1197·044 (1171·822–1282·061)	100%	0·910 (0·849–0·983)	100%	1196·134 (1170·910–1281·129)	100%
**2021**						
Rotavirus	108·470 (95·982–130·976)	24·4% (21·6–29·5)	0·148 (0·132–0·167)	30·3% (26·9–34·1)	108·322 (95·832–130·823)	24·4% (21·6–29·5)
*Shigella* sp	36·099 (32·301–42·694)	8·1% (7·3–9·6)	0·017 (0·014–0·020)	3·4% (2·8–4·0)	36·082 (32·285–42·678)	8·1% (7·3–9·6)
Norovirus GII	32·870 (28·514–39·967)	7·4% (6·4–9·0)	0·084 (0·069–0·101)	17·1% (14·1–20·6)	32·786 (28·435–39·882)	7·4% (6·4–9·0)
Adenovirus 40/41	32·722 (28·172–40·141)	7·4% (6·3–9·0)	0·040 (0·034–0·047)	8·2% (6·9–9·7)	32·682 (28·131–40·099)	7·4% (6·3–9·0)
ST-ETEC*	28·230 (23·530–35·704)	6·4% (5·3–8·0)	..	..	28·230 (23·530–35·704)	6·4% (5·3–8·1)
*Cryptosporidium* sp*	25·789 (21·782–31·922)	5·8% (4·9–7·2)	..	..	25·789 (21·782–31·922)	5·8% (4·9–7·2)
*C jejuni* or *C coli*	24·272 (20·883–29·797)	5·5% (4·7–6·7)	0·032 (0·026–0·039)	6·5% (5·4–8·0)	24·240 (20·852–29·764)	5·5% (4·7–6·7)
Astrovirus	21·263 (17·960–26·982)	4·8% (4·0–6·1)	0·019 (0·016–0·022)	3·9% (3·3–4·5)	21·244 (17·942–26·963)	4·8% (4·0–6·1)
Sapovirus*	20·684 (18·029–25·212)	4·7% (4·1–5·7)	..	..	20·684 (18·029–25·212)	4·7% (4·1–5·7)
Typical EPEC*	16·353 (13·111–21·477)	3·7% (3·0–4·8)	..	..	16·353 (13·111–21·477)	3·7% (3·0–4·8)
*Salmonella* sp	7·945 (6·752–9·977)	1·8% (1·5–2·2)	0·026 (0·023–0·030)	5·4% (4·6–6·2)	7·919 (6·726–9·950)	1·8% (1·5–2·2)
*V cholerae*	6·033 (5·026–7·887)	1·4% (1·1–1·8)	0·000 (0·000–0·000)	0·0% (0·0–0·0)	6·033 (5·026–7·887)	1·4% (1·1–1·8)
Other	83·103 (71·416–101·964)	18·7% (16·1–23·0)	0·123 (0·108–0·141)	25·2% (22·0–28·7)	82·980 (71·293–101·839)	18·7% (16·1–23·0)
Total	443·833 (390·833–539·647)	100%	0·490 (0·437–0·546)	100%	443·342 (390·357–539·150)	100%

Data are mean (95% CI). EPEC=enteropathogenic *Escherichia coli*. ST-ETEC=heat-stable, toxin-producing, enterotoxigenic *E coli*.

* Not estimated for high-income countries.

The cause with the largest estimated global attribution was rotavirus. In LMICs, the proportion of deaths from diarrhoea in children younger than 5 years estimated to be attributable to rotavirus appeared lower in 2021 (108 322 [24·4%] of 443 342, 95% uncertainty interval 21·6–29·5) than in 2000 (316 382 [26·5%] of 1 196 134, 25·7–28·5; table 1), but the 95% CIs overlapped. Of the six WHO regions, deaths from diarrhoea estimated to be attributable to rotavirus ranged from 6730 (20·2%) of 33 350 in the region of the Americas to 44 530 (33·0%) of 135 080 in the Eastern Mediterranean region in 2000 and from 1100 (18·1%) of 6070 in the region of the Americas to 17 470 (31·4%) of 55 720 in the Eastern Mediterranean region in 2021 (table 2). Comparing 2000 and 2021, deaths from diarrhoea estimated to be attributable to rotavirus declined the most in the European region (11·1%), the Western Pacific region (10·0%), the region of the Americas (9·9%), the South-East Asia region (5·2%), the Eastern Mediterranean region (4·8%), and the African region (4·2%). In 2000, the second largest estimated global attribution was norovirus GII (table 1); the proportion of deaths from diarrhoea in children younger than 5 years estimated to be attributable to norovirus GII was 95 817 (8·0%) of 1 196 134 in LMICs and 225 (24·7%) of 910 in HICs. In 2021, *Shigella* sp had the second largest estimated attribution in LMICs (36 082 [8·1%] of 443 342), but norovirus remained with the second largest estimated attribution in HICs (84 [17·1%] of 490). Estimated attribution to *Salmonella* sp was higher in HICs and to *Shigella* sp was lower in HICs than in LMICs in both years (table 1). Comparing estimates for regions in 2021, *Shigella* sp, sapovirus, and *V cholerae* had the highest attribution in the South-East Asia region; norovirus had the highest attribution in the region of the Americas; *Cryptosporidium* sp, typical EPEC, and ST-ETEC had the highest attribution in the African region; and *C jejuni* or *C coli* and astrovirus had the highest attribution in the Eastern Mediterranean region (table 2).

**Table 2:** Estimated proportion of deaths from diarrhoea in children younger than 5 years by cause for each WHO region in 2000 and 2021

	**African region**	**Region of the Americas**	**South-East Asia region**	**Eastern Mediterranean region**	**Western Pacific region**	**European region**
**2000**						
Rotavirus	23·8% (22·8 to 25·5)	20·2% (17·4 to 21·1)	28·9% (27·4 to 30·7)	33·0% (30·9 to 35·3)	26·9% (25·6 to 28·4)	23·5% (22·1 to 24·9)
Norovirus GII	7·3% (5·9 to 8·8)	16·6% (12·8 to 18·8)	9·2% (8·0 to 10·5)	5·4% (3·8 to 7·1)	9·5% (8·2 to 10·9)	7·8% (6·6 to 9·3)
Astrovirus	5·4% (4·0 to 7·1)	3·6% (2·5 to 4·7)	8·5% (7·0 to 9·9)	12·3% (9·8 to 14·9)	4·7% (3·7 to 5·8)	4·0% (3·1 to 5·3)
*Shigella* sp	5·0% (4·3 to 5·8)	7·4% (5·9 to 8·6)	8·8% (7·6 to 9·9)	7·6% (6·3 to 9·0)	6·4% (5·5 to 7·6)	5·1% (4·2 to 6·0)
Adenovirus 40/41	6·9% (5·5 to 9·0)	6·9% (5·2 to 8·5)	6·5% (5·4 to 7·7)	5·8% (4·2 to 7·3)	4·4% (3·4 to 5·5)	4·7% (3·7 to 5·7)
Sapovirus	4·7% (3·7 to 6·1)	3·3% (2·4 to 4·8)	9·6% (8·2 to 10·8)	4·3% (2·2 to 6·2)	3·1% (2·3 to 4·0)	3·5% (2·8 to 4·5)
*Campylobacter jejuni* or *Campylobacter coli*	4·4% (3·8 to 5·4)	3·3% (2·5 to 3·8)	4·6% (3·6 to 5·6)	14·3% (11·9 to 16·8)	1·9% (1·4 to 2·7)	3·9% (3·1 to 4·6)
ST-ETEC	7·8% (5·8 to 9·8)	2·1% (1·5 to 3·4)	2·7% (2·0 to 3·4)	3·2% (2·3 to 4·3)	2·6% (1·7 to 3·4)	2·4% (1·9 to 3·2)
*Cryptosporidium* sp	7·0% (5·6 to 8·5)	2·2% (1·6 to 2·7)	3·1% (2·5 to 3·9)	3·6% (2·6 to 4·7)	2·8% (2·0 to 3·6)	2·5% (2·1 to 3·0)
Typical EPEC	4·7% (3·4 to 6·2)	1·2% (0·8 to 1·6)	1·7% (1·3 to 2·3)	2·0% (1·4 to 2·7)	1·5% (1·1 to 2·1)	1·4% (1·1 to 1·9)
*Vibrio cholerae*	1·5% (0·9 to 2·2)	0·0% (−0·9 to 5·9)	4·9% (4·0 to 5·6)	2·0% (1·3 to 2·8)	1·0% (0·4 to 1·5)	1·4% (1·0 to 2·1)
*Salmonella* sp	1·7% (1·3 to 2·1)	2·3% (1·5 to 3·1)	1·4% (0·9 to 2·0)	2·6% (1·8 to 3·4)	1·6% (1·0 to 2·3)	2·0% (1·5 to 2·6)
Other	19·8% (17·3 to 22·6)	30·9% (25·1 to 32·4)	10·3% (8·9 to 11·7)	4·0% (2·0 to 5·8)	33·7% (30·9 to 36·1)	37·7% (34·7 to 40·6)
**2021**						
Rotavirus	22·8% (21·9 to 24·2)	18·1% (16·4 to 19·1)	27·4% (25·9 to 29·4)	31·4% (28·5 to 33·9)	24·2% (22·8 to 25·4)	20·9% (19·3 to 22·6)
*Shigella* sp	6·4% (5·5 to 7·4)	10·7% (8·6 to 12·1)	13·7% (11·5 to 16·1)	12·5% (9·0 to 15·3)	5·4% (4·6 to 6·4)	8·8% (6·6 to 11·4)
Norovirus GII	7·2% (6·0 to 8·5)	15·5% (13·1 to 17·5)	9·4% (8·4 to 10·6)	5·3% (3·6 to 7·0)	9·3% (8·1 to 10·3)	7·3% (6·3 to 8·5)
Adenovirus 40/41	7·5% (6·3 to 8·8)	7·7% (6·2 to 9·0)	7·7% (6·2 to 9·5)	7·0% (4·3 to 9·4)	3·9% (3·1 to 4·9)	5·5% (4·1 to 7·0)
ST-ETEC	7·8% (5·9 to 9·4)	2·1% (1·5 to 3·1)	2·7% (2·0 to 3·7)	3·3% (2·4 to 4·5)	2·3% (1·5 to 2·9)	2·3% (1·8 to 3·3)
*Cryptosporidium* sp	6·9% (5·7 to 8·1)	2·1% (1·6 to 2·7)	3·1% (2·4 to 4·0)	3·6% (2·6 to 4·9)	2·5% (1·8 to 3·1)	2·4% (1·9 to 3·1)
*C jejuni* or *C coli*	4·3% (3·8 to 5·1)	3·1% (2·3 to 3·6)	4·5% (3·5 to 5·6)	14·1% (11·7 to 16·9)	1·8% (1·3 to 2·5)	3·6% (2·9 to 4·5)
Astrovirus	4·2% (3·4 to 5·1)	2·4% (1·8 to 3·1)	4·9% (3·9 to 6·6)	8·9% (6·0 to 12·1)	3·0% (2·2 to 4·0)	2·4% (1·8 to 3·3)
Sapovirus	4·3% (3·5 to 5·3)	3·0% (2·1 to 3·9)	7·8% (6·5 to 9·3)	4·1% (2·2 to 6·2)	2·1% (1·6 to 2·8)	3·2% (2·4 to 4·1)
Typical EPEC	4·5% (3·4 to 5·9)	1·1% (0·7 to 1·4)	1·6% (1·2 to 2·1)	1·9% (1·3 to 2·5)	1·4% (0·9 to 2·0)	1·3% (1·0 to 1·8)
*Salmonella* sp	1·7% (1·3 to 2·0)	2·1% (1·5 to 2·9)	1·5% (0·9 to 2·1)	2·4% (1·8 to 3·2)	1·7% (1·1 to 2·3)	1·8% (1·4 to 2·3)
*V cholerae*	1·1% (0·8 to 1·6)	0·0% (−0·6 to 3·7)	3·0% (2·2 to 4·1)	1·3% (0·8 to 2·0)	0·8% (0·4 to 1·2)	0·8% (0·5 to 1·2)
Other	21·2% (18·9 to 24·1)	32·0% (28·2 to 34·7)	12·8% (10·9 to 14·6)	4·2% (2·0 to 6·2)	41·8% (38·1 to 44·7)	39·7% (34·2 to 44·5)

Data are mean (95% CI). EPEC=enteropathogenic *Escherichia coli*. ST-ETEC=heat-stable, toxin-producing, enterotoxigenic *E coli*.

The number of estimated global deaths from diarrhoea in children younger than 5 years globally decreased from 1·197 million (95% CI 1·172–1·282) in 2000 to 0·444 million (0·391–0·540) in 2021, a decline of 62·9% (table 1). The percentage decline was the same for LMICs, where more than 99% of these deaths occur. In HICs, deaths from diarrhoea declined from 910 to 490, a reduction of 46·1%. Each of the LMIC regions had declines in deaths from diarrhoea (table 3).

**Table 3:** Estimated number of deaths from diarrhoea in children younger than 5 years, in thousands, by cause for each WHO region in 2000 and 2021

		**African region**	**Region of the Americas**	**South-East Asia region**	**Eastern Mediterranean region**	**Western Pacific region**	**European region**
2000							
	Rotavirus	140·10 (125·68–156·19)	6·73 (5·66–7·16)	108·01 (97·75–121·86)	44·53 (37·83–58·57)	14·37 (11·04–28·20)	2·52 (2·15–3·11)
	Norovirus GII	42·81 (34·26–52·34)	5·53 (4·21–6·35)	34·21 (28·44–40·05)	7·32 (4·71–9·46)	5·06 (3·76–10·10)	0·84 (0·70–1·07)
	Astrovirus	32·01 (23·29–41·77)	1·18 (0·85–1·55)	31·92 (25·86–38·49)	16·58 (12·58–21·10)	2·49 (1·70–5·06)	0·43 (0·30–0·62)
	*Shigella* sp	29·32 (24·17–35·07)	2·47 (1·95–2·85)	32·73 (27·21–38·87)	10·26 (8·20–13·49)	3·40 (2·44–7·06)	0·55 (0·45–0·69)
	Adenovirus 40/41	40·65 (32·16–54·74)	2·31 (1·66–2·82)	24·10 (19·45–29·19)	7·80 (5·55–10·55)	2·35 (1·55–5·16)	0·50 (0·40–0·66)
	Sapovirus	27·85 (21·74–36·34)	1·11 (0·77–1·55)	35·79 (28·59–41·32)	5·78 (3·13–8·48)	1·64 (1·05–3·53)	0·38 (0·30–0·51)
	*Campylobacter jejuni* or *Campylobacter coli*	25·99 (21·36–31·88)	1·10 (0·80–1·29)	17·08 (13·17–21·28)	19·38 (15·71–24·21)	1·04 (0·64–2·26)	0·42 (0·33–0·54)
	ST-ETEC	46·03 (33·96–59·98)	0·71 (0·49–1·19)	9·93 (7·44–12·92)	4·30 (2·95–6·17)	1·36 (1·26–2·99)	0·25 (0·20–0·35)
	*Cryptosporidium* sp	41·16 (33·38–50·20)	0·74 (0·55–0·88)	11·58 (8·44–15·80)	4·81 (3·50–6·76)	1·52 (1·51–3·37)	0·27 (0·21–0·35)
	Typical EPEC	27·66 (19·06–36·45)	0·40 (0·27–0·53)	6·36 (4·19–9·06)	2·66 (1·86–3·89)	0·82 (0·50–1·80)	0·15 (0·12–0·22)
	*Vibrio cholerae*	8·67 (5·68–13·48)	0·00 (0·00–1·94)	18·13 (14·75–21·74)	2·73 (1·75–4·05)	0·51 (0·36–1·39)	0·15 (0·10–0·23)
	*Salmonella* sp	10·26 (7·77–13·05)	0·76 (0·51–1·03)	5·22 (3·45–7·23)	3·50 (2·55–4·72)	0·87 (0·81–1·86)	0·21 (0·16–0·28)
	Other	116·89 (96·07–137·37)	10·30 (8·36–11·18)	38·58 (31·92–45·10)	5·43 (2·82–8·18)	17·98 (13·91–34·59)	4·05 (3·47–4·85)
	Total	589·42 (530·68–649·15)	33·35 (31·08–36·18)	373·63 (333·09–413·32)	135·08 (117·62–171·74)	53·42 (41·69–104·99)	10·74 (9·47–12·73)
2021							
	Rotavirus	71·42 (55·07–103·49)	1·10 (0·78–1·84)	15·25 (12·44–18·78)	17·47 (11·67–34·55)	2·76 (1·89–4·18)	0·30 (0·17–0·52)
	*Shigella* sp	20·14 (15·23–29·64)	0·65 (0·42–1·14)	7·60 (5·60–9·70)	6·95 (4·65–11·74)	0·61 (0·45–0·98)	0·13 (0·06–0·27)
	Norovirus GII	22·51 (16·81–35·22)	0·94 (0·66–1·45)	5·21 (3·79–6·48)	2·95 (1·83–5·42)	1·06 (0·72–1·66)	0·10 (0·06–0·18)
	Adenovirus 40/41	23·50 (16·99–33·65)	0·47 (0·35–0·75)	4·28 (3·26–6·18)	3·90 (2·17–7·36)	0·45 (0·29–0·73)	0·08 (0·04–0·15)
	ST-ETEC	24·45 (17·88–38·60)	0·13 (0·08–0·20)	1·51 (1·08–2·11)	1·84 (1·14–3·82)	0·26 (0·17–0·43)	0·03 (0·02–0·06)
	*Cryptosporidium* sp	21·60 (16·53–33·56)	0·13 (0·08–0·22)	1·72 (1·26–2·34)	2·02 (1·16–4·19)	0·28 (0·17–0·54)	0·04 (0·02–0·08)
	*C jejuni* or *C coli*	13·47 (10·22–19·06)	0·19 (0·12–0·31)	2·49 (1·89–3·29)	7·84 (5·12–15·87)	0·20 (0·12–0·36)	0·05 (0·03–0·09)
	Astrovirus	13·01 (8·98–21·16)	0·14 (0·09–0·27)	2·72 (1·99–3·76)	4·99 (2·83–11·63)	0·34 (0·23–0·60)	0·03 (0·02–0·07)
	Sapovirus	13·54 (9·95–21·61)	0·18 (0·10–0·32)	4·36 (3·35–5·70)	2·31 (1·23–4·82)	0·24 (0·17–0·44)	0·05 (0·02–0·09)
	Typical EPEC	14·15 (8·48–22·75)	0·07 (0·05–0·11)	0·90 (0·67–1·39)	1·06 (0·61–2·72)	0·15 (0·11–0·30)	0·02 (0·01–0·04)
	*Salmonella* sp	5·39 (3·78–8·26)	0·13 (0·08–0·23)	0·81 (0·49–1·20)	1·36 (0·79–2·63)	0·20 (0·12–0·34)	0·03 (0·01–0·05)
	*V cholerae*	3·57 (2·18–5·94)	0·00 (0·00–0·26)	1·65 (1·20–2·30)	0·71 (0·35–1·67)	0·09 (0·04–0·18)	0·01 (0·01–0·02)
	Other	66·27 (48·29–95·69)	1·95 (1·42–2·81)	7·10 (5·50–9·53)	2·31 (1·20–5·11)	4·76 (3·34–7·70)	0·57 (0·37–0·92)
	Total	313·03 (249·07–453·84)	6·07 (4·53–9·91)	55·61 (45·36–68·26)	55·72 (36·97–109·54)	11·41 (7·98–18·00)	1·44 (0·86–2·46)
Total decline		46·9%	83·7%	85·1%	58·8%	78·6%	84·1%

Data are mean (95% CI). EPEC=enteropathogenic *Escherichia coli*. ST-ETEC=heat-stable, toxin-producing, enterotoxigenic *Escherichia coli*.

**Figure 2:**
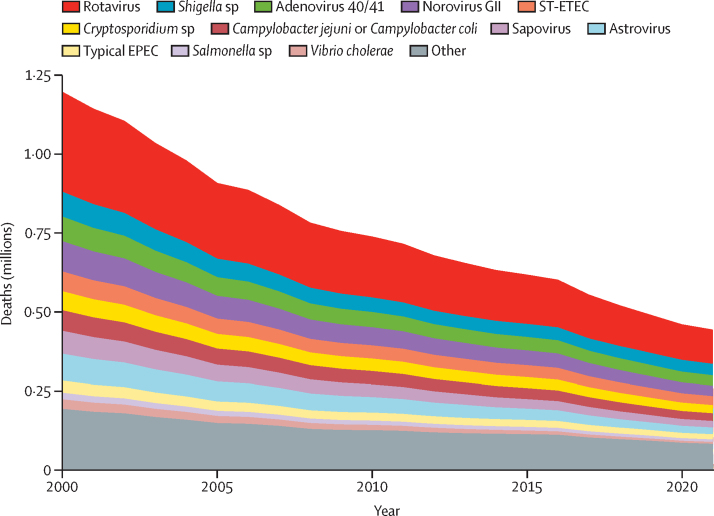
Estimated global number of deaths from diarrhoea in children younger than 5 years by cause during 2000–21 EPEC=enteropathogenic *Escherichia coli*. ST-ETEC=heat-stable, toxin-producing enterotoxigenic *E coli*.

All causes declined in the number of deaths from diarrhoea in children younger than 5 years from 2000 to 2021 (figure 2). The estimated global number of deaths attributable to rotavirus declined from 316 634 (95% CI 307 845–341 341) in 2000 to 108 470 (95 982–130 976) in 2021, a 65·7% reduction (table 1). Regionally, in LMICs, deaths attributable to rotavirus were estimated to be reduced by 88·1% in the European region, 85·9% in the South-East Asia region, 83·7% in the region of the Americas, 80·8% in the Western Pacific region, 60·8% in the Eastern Mediterranean region, and 49·0% in the African region. In 2021, these two regions had 88 890 (81·9%) of 108 470 global rotavirus deaths and the South-East Asia region had 15 250 (14·1%; table 3). The estimated global number of deaths attributable to norovirus declined by 65·8% during 2000–21. For other causes, declines in the numbers of deaths ranged from 54·2% for *Shigella* sp to 80·0% for *V cholerae* (table 1). Deaths from cholera in children younger than 5 years decreased by 90·9% in the South-East Asia region and by 58·8% in the African region (table 3).

## Discussion

We could attribute a cause to 81·3% of global deaths from diarrhoea in children younger than 5 years in 2021, similar to the percentage attribution (72·6%) in the GPDS network, which assessed the causes of diarrhoea in children younger than 5 years who were admitted to hospital via qPCR diagnostic methods, whereas we adjusted published results on causes when necessary to account for the poor sensitivity of non-qPCR methods.^10^ The GPDS study used methods derived from children with diarrhoea and matched-control individuals in GEMS^6^ and MAL-ED^7^ to estimate attributable fractions. We used the same methods to estimate attributable fractions from published studies that did not have control individuals.

The attribution of rotavirus to deaths from diarrhoea was the largest of the 12 causes but might have declined from 26**·**5% in 2000 to 24·4% in 2021. These proportions are consistent with the finding of 27·8% for deaths attributable to rotavirus in 2011 using different methods (appendix 1 p 22).^5^ The number of deaths from diarrhoea estimated to be attributable to rotavirus decreased by two-thirds during 2020–21. The introduction of a rotavirus vaccine in some countries in each region probably contributed to the reduction of deaths attributable to rotavirus and could partly explain the reductions of 83·7–88·1% in the region of the Americas, the South-East Asia region, and the European region. Incomplete vaccine coverage and reduced efficacy of available vaccines in high-mortality settings probably restricted the effect in other regions.^4^ Rotavirus vaccination was estimated to have prevented 15% of rotavirus deaths in 2019, when global vaccine coverage was 40%;^17^ our analyses suggested that the vaccines prevented 9% of rotavirus deaths in that year.

For norovirus, our estimates of 8·0% in 2000 and 7·4% in 2021 are less than a previous estimate of 9·9% for 2011,^5^ possibly because we only included norovirus Gll in this systematic review and Bayesian multinomial analysis. For *Shigella* sp, the estimates of 6·6% for 2000 and 8·1% for 2021 are higher than a previous estimate of 3·9%,^5^ probably related to our adjustment for the improved sensitivity of qPCR methods. Most causes were estimated to be similar to previous estimates,^5^ but ST-ETEC and typical EPEC were lower. For ST-ETEC, we only included heat-stable toxin-producing bacteria, eliminating *E coli* that produce only heat-labile toxin. We included only typical EPEC and not other potentially pathogenic *E coli*.^18^ We did not include *Giardia lamblia* because it has not been shown to have a significant attributable fraction for moderate-to-severe diarrhoea.^6^

The studies of endemic childhood diarrhoea that we used for our estimates probably underestimated deaths due to cholera outbreaks.^19^, ^20^ Countries in the region of the Americas had large cholera outbreaks in the 1990s, but these had subsided by 2000.^21^ Haiti had a cholera outbreak after the earthquake in 2010; illnesses with or deaths from cholera were not reported in 2021, but resurged in 2022.^22^ Because we have reported the estimated causes of deaths from diarrhoea in the specific years 2000 and 2021, the region of the Americas was not estimated to have cholera deaths. Many countries in sub-Saharan Africa have had cholera outbreaks since 2010.^23^

Our estimates are substantially different from those of the Global Burden of Disease Study (GBD) 2019 (appendix 1 p 22).^24^ Comparison is difficult because GBD might attribute the death from diarrhoea to more than one pathogen, resulting in larger percentage attributions for many causes than our estimates and larger total attributions than the actual number of deaths reported by GBD.^24^ Our methods, a multinomial model that required causal fractions to total 1, attributed each death to a single cause. This approach has been used for previous estimates of the causes of death in children younger than 5 years.^1^ GBD also attributes each death to a single cause, but does not use that method for assigning causes of deaths from diarrhoea, instead allowing multiple causes for each death.

One strength of our analysis is the systematic review of studies of the causes of diarrhoea in children hospitalised for severe illness. We did not include studies of single diarrhoea pathogens because these studies provide a biased estimate of the importance of that pathogen.^5^ Because most of the publications on the causes of diarrhoea are case series without control individuals and because asymptomatic infection in LMICs is common, our estimates needed to be adjusted for attributable fractions via evidence from landmark studies of the causes of diarrhoea that included control individuals. Furthermore, because we needed to include publications in the past two decades to assess trends and have sufficient data for analysis, we developed an adjustment for results on the basis of previous pathogen-detection methods to reflect current state-of-the-art qPCR methods.

The limitations of our analysis are primarily related to data availability and applicability of these data to deaths from diarrhoea. Although the search for data resulted in a substantial number of studies, our results have incomplete representation of geographical areas and trends in causes during two decades. For this reason, we chose to present results only at the regional level. Because there is little information on the causes of diarrhoea resulting in death, the causes of diarrhoea in children with illness that was sufficiently severe to require hospitalisation were used as proxies for those of deaths.^5^, ^22^ Increasing access to and quality of care could change the rate of survival of severe diarrhoea; this rate could differ for specific causes. We used several commonly used covariates to estimate the associations between socioeconomic and health-service conditions in countries and the distribution of diarrhoea causes. Other measures, such as access to or quality of care, might be more strongly related to cause than the standard covariates we used. We chose not to use pathogen-specific case-fatality risk because such data are scarce and can be biased.^25^ We found only one study in the review that examined causes in children who died from diarrhoea. We did not include these data in our model because our focus was on children admitted to hospital, but the results provide a comparison with our estimates of the relative importance of the different causes. A prospective, hospital-based study in Venezuela of deaths from diarrhoea in children younger than 5 years found that rotavirus was associated with 21% of deaths and *Shigella* sp was associated with 19% of deaths; calicivirus, *Salmonella* sp, and *Campylobacter* sp were each associated with 3–6% of deaths.^26^ Studies from 2015 onwards in multiple countries with minimally invasive tissue sampling for childhood deaths could help to assess the roles of malnutrition and specific infection in deaths from diarrhoea.^27^ Studies of sensitive diagnostic methods, such as qPCR, often find more than one diarrhoeagenic pathogen in the faeces of people with severe diarrhoea. Our use of attributable fractions adjusts for the occurrence of asymptomatic infections with these potential pathogens; however, that current methods do not permit the assessment of whether specific co-infections might result in enhanced severity and lethality is a limitation.

Preventing the 444 000 global estimated deaths from diarrhoea in children younger than 5 years that remained in 2021 should be a priority. Our finding that rotavirus was estimated to have caused a quarter of these deaths, even after substantial declines in the number of rotavirus deaths, suggests that it needs more attention, including increased use of existing rotavirus vaccines, development of vaccines with increased protection in high-mortality settings, and improved access to and use of effective treatments. We found that another 11 pathogens were each estimated to result in less than 10% of deaths from diarrhoea in children younger than 5 years in 2021. Vaccines against these pathogens are likely to be highly specific and result in only small overall reductions in endemic deaths from diarrhoea. The use of cholera vaccines might be important in some settings for the prevention and interruption of epidemics. Justification for vaccines against the other 10 pathogens might need to be based on the prevention of morbidity and reduction of treatment costs for this age group and others.^19^ Increased use of rotavirus and cholera vaccines could aid further reductions in deaths from diarrhoea, alongside general preventive measures to interrupt faecal–oral transmission of enteropathogens and access to effective treatment for all diarrhoeal illnesses. Further improvements are needed for continued reduction of deaths from diarrhoea in children younger than 5 years.

## Contributors

## Data sharing

All input data and scripts related to the statistical analysis are available with publication, without restriction, at https://github.com/jamieperin/diarrhoea_etiology. No other documents will be available.

## Acknowledgments

## Declaration of interests

We declare no competing interests.

## Supplementary Materials

Supplementary appendix 1

Equitable Partnership Declaration
