# Supplementary material for: Estimated global and regional causes of deaths from diarrhoea in children younger than 5 years during 2000–21: a systematic review and Bayesian multinomial analysis
Source: Lancet Glob Health. 2024 Apr 20;12(6):e919–28. doi: 10.1016/S2214-109X(24)00078-0 (PMC11099298; doi:10.1016/S2214-109X(24)00078-0)
Supplement: Supplementary appendix 1 [file mmc1.pdf]

THE LANCET
Global Health

Supplementary appendix 1

This appendix formed part of the original submission and has been peer reviewed. We post it as supplied by the authors.

Supplement to: Black RE, Perin J, Yeung D, et al. Estimated global and regional causes of deaths from diarrhoea in children younger than 5 years during 2000–21: a systematic review and Bayesian multinomial analysis. *Lancet Glob Health* 2024; published online April 19. https://doi.org/10.1016/S2214-109X(24)00078-0.

Contents

Web Appendix Full search strategy (Pubmed) 2

Web Appendix PRIMSA Checklist 2020 3

Web appendix Figure 1. Location of included studies 6

Web Appendix Table 1. Description of study (input) data by country income level 7

Web Appendix Table 2. Choice of GEMS or MAL-ED datasets for scalar derivation for each pathogen 7

Web Appendix Table 3. Description of diagnostic methods in study (input) data 8

Web Appendix Figure 2. Comparison of before and after scalars 9

Web Appendix Statistical Methods. Details related to statistical methods 11

Web Appendix Figure 3. Model coefficient estimates 13

Web Appendix Figure 4. Convergence for high income model 15

Web Appendix Figure 5. Convergence for low and middle-income model. 18

Web Appendix Table 4. Comparison of Previous[6,11,25] and current estimates of the etiologies of diarrhea deaths 22

Web Appendix Full search strategy (Pubmed)

Search Group 1 (child) AND Search Group 2 (diarrhea) AND Search Group 3 (hospital)

1. Child
   "infant"[mh] OR "Infant, Newborn"[mh] OR "Infant, Premature"[mh] OR "Child"[mh] OR "Child, Preschool"[mh] OR "Minors"[mh] OR "infant"[tw] "infants"[tw] OR "neonate"[tw] OR "neonates"[tw] OR "neonatal"[tw] OR "newborn"[tw] OR "newborns"[tw] OR "new-born"[tw] OR "new-borns"[tw] OR "babies"[tw] OR "baby"[tw] OR "Premature"[tw] OR "preterm"[tw] OR "pre term"[tw] OR "child"[tw] OR "children"[tw] OR "youth"[tw] OR "youths"[tw] OR "young people"[tw] OR "childhood"[tw] OR "toddler"[tw] OR "toddlers"[tw] OR "kid"[tw] OR "kids"[tw] OR "young patient"[tw] OR "young patients"[tw] OR "boy"[tw] OR "boys"[tw] OR "girl"[tw] OR "girls"[tw] OR "young age"[tw] OR "pediatric"[tw] OR "pre-schooler"[tw] OR "preschooler"[tw] OR "under 5"[tw] OR "under five"[tw] OR "under fives"[tw] OR "less than five"[tw] OR "perinatal"[tw]

2. Diseases
   "Diarrhea"[mh] OR "gastroenteritis"[mh] OR "Cholera"[mh] OR "dysentery"[mh] OR "Rotavirus"[mh] OR "Escherichia coli"[mh] OR "Salmonella"[mh] OR "Shigella"[mh] OR "Campylobacter"[mh] OR "Giardia lamblia"[mh] OR "Vibrio cholerae"[mh] OR "Cryptosporidium"[mh] OR "Norwalk virus"[mh] OR "Avastrovirus"[mh] OR "Coronavirus"[mh] OR "Adenoviridae"[mh] OR "Sapovirus"[mh] OR "Aeromonas"[mh] OR "Diarrhea"[tw] OR "Diarrheas"[tw] OR "Diarrheal"[tw] OR "Diarrhoeal"[tw] OR "Diarrhoea"[tw] OR "Diarrhoeas"[tw] OR "Dysentery"[tw] OR "Dysenteria"[tw] OR "enteritis"[tw] OR "enteritides"[tw] OR "Cholera"[tw] OR "Choleras"[tw] OR "vibrioCholerae"[tw] OR "gastroenteritis"[tw] OR "gastroenteritides"[tw] OR "gastroduodenitis"[tw] OR "gastrointestinal acute infection"[tw] OR "gastrointestinal acute infections"[tw] OR "digestive tract infections"[tw] OR "digestive infection"[tw] OR "gastrointestinal tract infection"[tw] OR "gastrointestine tract infection"[tw] OR "rotavirus"[tw] OR "Escherichia coli"[tw] OR "salmonella"[tw] OR "Shigella"[tw] OR "campylobacter"[tw] OR "giardia lamblia"[tw] OR "vibrio cholerae"[tw] OR "cryptosporidium"[tw] OR "entamoeba histolytica"[tw] OR "norovirus"[tw] OR "calicivirus"[tw] OR "norwalk agent"[tw] OR "astrovirus"[tw] OR "coronavirus"[tw] OR "adenovirus"[tw] OR "sapovirus"[tw] OR "aeromonas"[tw] OR "avastarovirus"[tw]

3. Hospital
   "Hospital"[mh] OR "Child, Hospitalized"[mh] OR "Hospitalization"[mh] OR "hospital"[tw] OR "in-patient"[tw] OR "hospitalization"[tw] OR "hospitalized"[tw]

Web Appendix PRIMSA Checklist 2020

Section and Topic	Item #	Checklist item	Location where item is reported
**TITLE**			
Title	1	Identify the report as a systematic review.	Title
**ABSTRACT**			
Abstract	2	See the PRISMA 2020 for Abstracts checklist.	Done
**INTRODUCTION**			
Rationale	3	Describe the rationale for the review in the context of existing knowledge.	Page 2
Objectives	4	Provide an explicit statement of the objective(s) or question(s) the review addresses.	P2
**METHODS**			
Eligibility criteria	5	Specify the inclusion and exclusion criteria for the review and how studies were grouped for the syntheses.	P3
Information sources	6	Specify all databases, registers, websites, organisations, reference lists and other sources searched or consulted to identify studies. Specify the date when each source was last searched or consulted.	P3
Search strategy	7	Present the full search strategies for all databases, registers and websites, including any filters and limits used.	Appendix
Selection process	8	Specify the methods used to decide whether a study met the inclusion criteria of the review, including how many reviewers screened each record and each report retrieved, whether they worked independently, and if applicable, details of automation tools used in the process.	P3
Data collection process	9	Specify the methods used to collect data from reports, including how many reviewers collected data from each report, whether they worked independently, any processes for obtaining or confirming data from study investigators, and if applicable, details of automation tools used in the process.	P3
Data items	10a	List and define all outcomes for which data were sought. Specify whether all results that were compatible with each outcome domain in each study were sought (e.g. for all measures, time points, analyses), and if not, the methods used to decide which results to collect.	P3
	10b	List and define all other variables for which data were sought (e.g. participant and intervention characteristics, funding sources). Describe any assumptions made about any missing or unclear information.	P3
Study risk of bias assessment	11	Specify the methods used to assess risk of bias in the included studies, including details of the tool(s) used, how many reviewers assessed each study and whether they worked independently, and if applicable, details of automation tools used in the process.	P3
Effect measures	12	Specify for each outcome the effect measure(s) (e.g. risk ratio, mean difference) used in the synthesis or presentation of results.	P3
Synthesis methods	13a	Describe the processes used to decide which studies were eligible for each synthesis (e.g. tabulating the study intervention characteristics and comparing against the planned groups for each synthesis (item #5)).	P3
	13b	Describe any methods required to prepare the data for presentation or synthesis, such as handling of missing summary statistics, or data conversions.	P3-5
	13c	Describe any methods used to tabulate or visually display results of individual studies and syntheses.	P5-6

	13d	Describe any methods used to synthesize results and provide a rationale for the choice(s). If meta-analysis was performed, describe the model(s), method(s) to identify the presence and extent of statistical heterogeneity, and software package(s) used.	P5-6
	13e	Describe any methods used to explore possible causes of heterogeneity among study results (e.g. subgroup analysis, meta-regression).	P5-6
	13f	Describe any sensitivity analyses conducted to assess robustness of the synthesized results.	Appendix
Reporting bias assessment	14	Describe any methods used to assess risk of bias due to missing results in a synthesis (arising from reporting biases).	P3
Certainty assessment	15	Describe any methods used to assess certainty (or confidence) in the body of evidence for an outcome.	P5-6
**RESULTS**			
Study selection	16a	Describe the results of the search and selection process, from the number of records identified in the search to the number of studies included in the review, ideally using a flow diagram.	P3, Figure 1, Appendix figure
	16b	Cite studies that might appear to meet the inclusion criteria, but which were excluded, and explain why they were excluded.	PRISMA figure
Study characteristics	17	Cite each included study and present its characteristics.	Not done
Risk of bias in studies	18	Present assessments of risk of bias for each included study.	Not done
Results of individual studies	19	For all outcomes, present, for each study: (a) summary statistics for each group (where appropriate) and (b) an effect estimate and its precision (e.g. confidence/credible interval), ideally using structured tables or plots.	Not done
Results of syntheses	20a	For each synthesis, briefly summarise the characteristics and risk of bias among contributing studies.	No done
	20b	Present results of all statistical syntheses conducted. If meta-analysis was done, present for each the summary estimate and its precision (e.g. confidence/credible interval) and measures of statistical heterogeneity. If comparing groups, describe the direction of the effect.	P6-7
	20c	Present results of all investigations of possible causes of heterogeneity among study results.	Tables 1-3
	20d	Present results of all sensitivity analyses conducted to assess the robustness of the synthesized results.	Appendix
Reporting biases	21	Present assessments of risk of bias due to missing results (arising from reporting biases) for each synthesis assessed.	Not done
Certainty of evidence	22	Present assessments of certainty (or confidence) in the body of evidence for each outcome assessed.	P6-7
**DISCUSSION**			
Discussion	23a	Provide a general interpretation of the results in the context of other evidence.	P7-9
	23b	Discuss any limitations of the evidence included in the review.	P8-9
	23c	Discuss any limitations of the review processes used.	P8-9
	23d	Discuss implications of the results for practice, policy, and future research.	P7-9
**OTHER INFORMATION**			
Registration and	24a	Provide registration information for the review, including register name and registration number, or state that the review was not	Not done

protocol		registered.	
	24b	Indicate where the review protocol can be accessed, or state that a protocol was not prepared.	Not done
	24c	Describe and explain any amendments to information provided at registration or in the protocol.	Not done
Support	25	Describe sources of financial or non-financial support for the review, and the role of the funders or sponsors in the review.	Gates, no role
Competing interests	26	Declare any competing interests of review authors.	None
Availability of data, code and other materials	27	Report which of the following are publicly available and where they can be found: template data collection forms; data extracted from included studies; data used for all analyses; analytic code; any other materials used in the review.	Data of individual studies and analytic code on github

From: Page MJ, McKenzie JE, Bossuyt PM, Boutron I, Hoffmann TC, Mulrow CD, et al. The PRISMA 2020 statement: an updated guideline for reporting systematic reviews. BMJ 2021;372:n71. doi: 10.1136/bmj.n71

For more information, visit: http://www.prisma-statement.org/

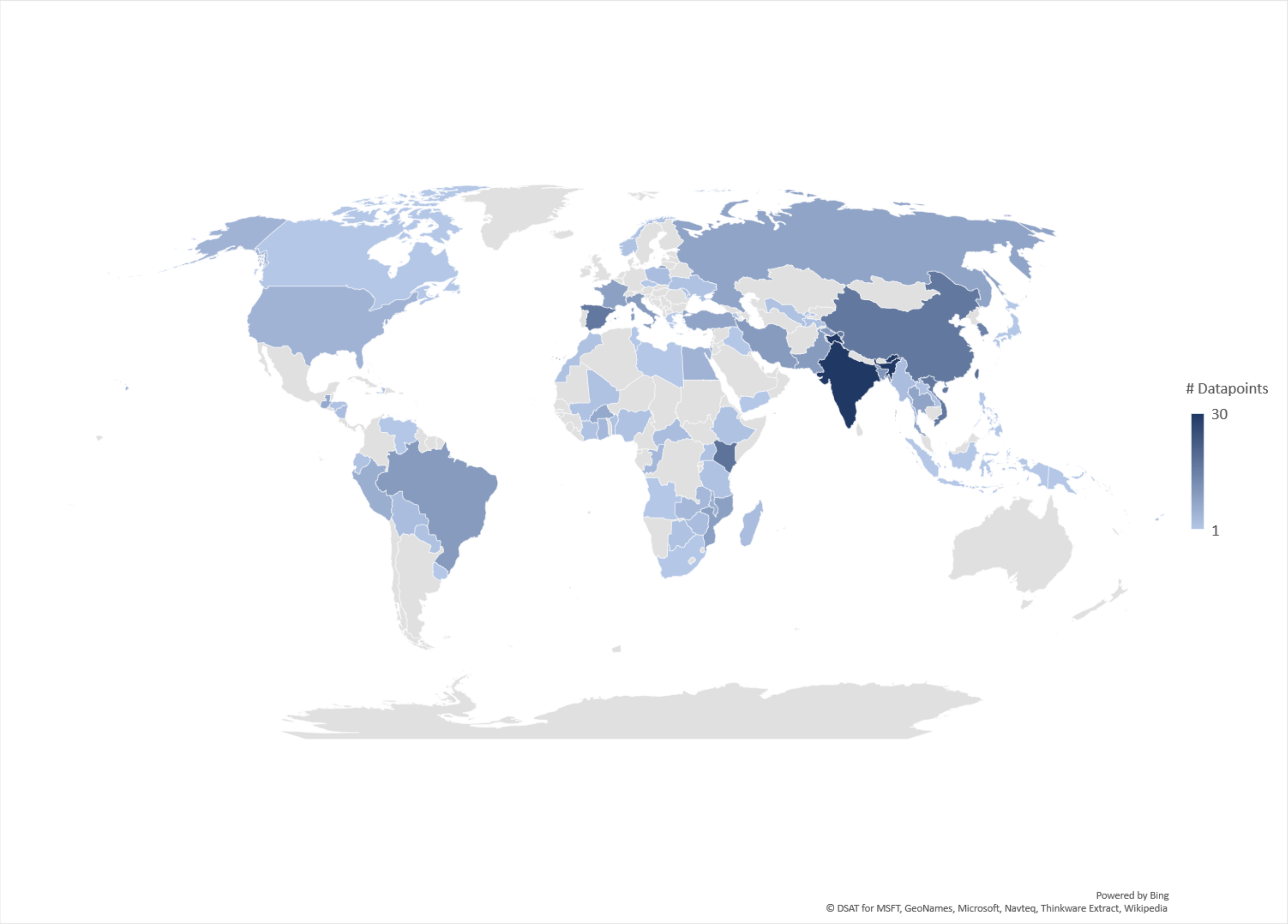

Web appendix Figure 1. Location of included studies

Web Appendix Table 1. Description of study (input) data by country income level

	Number of studies	Number of diarrhea cases
From High Income Countries	47	230088
From Low & Middle Income Countries	91	66194

Web Appendix Table 2. Choice of GEMS or MAL-ED datasets for scalar derivation for each pathogen

Pathogen	Dataset used for scalar	GEMS conventional diagnostic type	MAL-ED conventional diagnostic type	Notes
Adenovirus 40/41	MAL-ED	Nested EIA (adenovirus followed by adenovirus 40/41)	EIA	Direct detection by EIA is more representative
Astrovirus	MAL-ED	Endpoint PCR	EIA	EIA is more representative
Campylobacter jejuni/coli	GEMS	Culture	Culture	
Cryptosporidium sp	GEMS	EIA	EIA	
Norovirus GII	GEMS	Endpoint PCR	N/A (qPCR)	
Rotavirus	GEMS	EIA	EIA	
Salmonella sp	GEMS	Culture	Culture	
Sapovirus	GEMS	Endpoint PCR	Not tested	
Shigella sp	GEMS	Culture	Culture	
ST-ETEC	GEMS	Culture, picking of three *E. coli* colonies, and endpoint PCR for detection of STh	Culture, picking of five *E. coli* colonies, and endpoint PCR for detection of STh and STp	
Typical EPEC	GEMS	Culture, picking of three *E. coli* colonies, and endpoint PCR for detection of *bfpA*	Culture, picking of five *E. coli* colonies, and endpoint PCR for detection of *bfpA*	
Vibrio cholerae	GEMS	Culture	Culture	

Web Appendix Table 3. Description of diagnostic methods in study (input) data

		Diagnostic method – N (%) studies		
	**Total Studies reporting**	**Attributable fraction**	**Conventional**	**qPCR**
Rotavirus	120*	4 (3%)	96 (80%)	19 (16%)
Norovirus GII	47	3(6%)	16 (34%)	28 (60%)
Adenovirus 40/41	26	3 (12%)	14 (54%)	9 (35%)
ST-ETEC	11	4 (36%)	1 (9%)	6 (55%)
Shigella sp	56	2 (4%)	44 (79%)	10 (18%)
Cryptosporidium sp	25	4 (16%)	14 (56%)	7 (28%)
Sapovirus	34	3 (9%)	17 (50%)	14 (41%)
Astrovirus	60	3 (5%)	45 (75%)	12 (2%)
Campylobacter jejuni/coli	53	3 (6%)	39 (74%)	11 (21%)
Typical EPEC	13	4 (31%)	4 (31%)	5 (38%)
Vibrio cholerae	20	2 (10%)	12 (60%)	6 (30%)
Salmonella sp	65*	3 (5%)	49 (75%)	12 (18%)

*includes a study where the diagnostic method was not specified

Web Appendix Figure 2. Comparison of before and after scalars

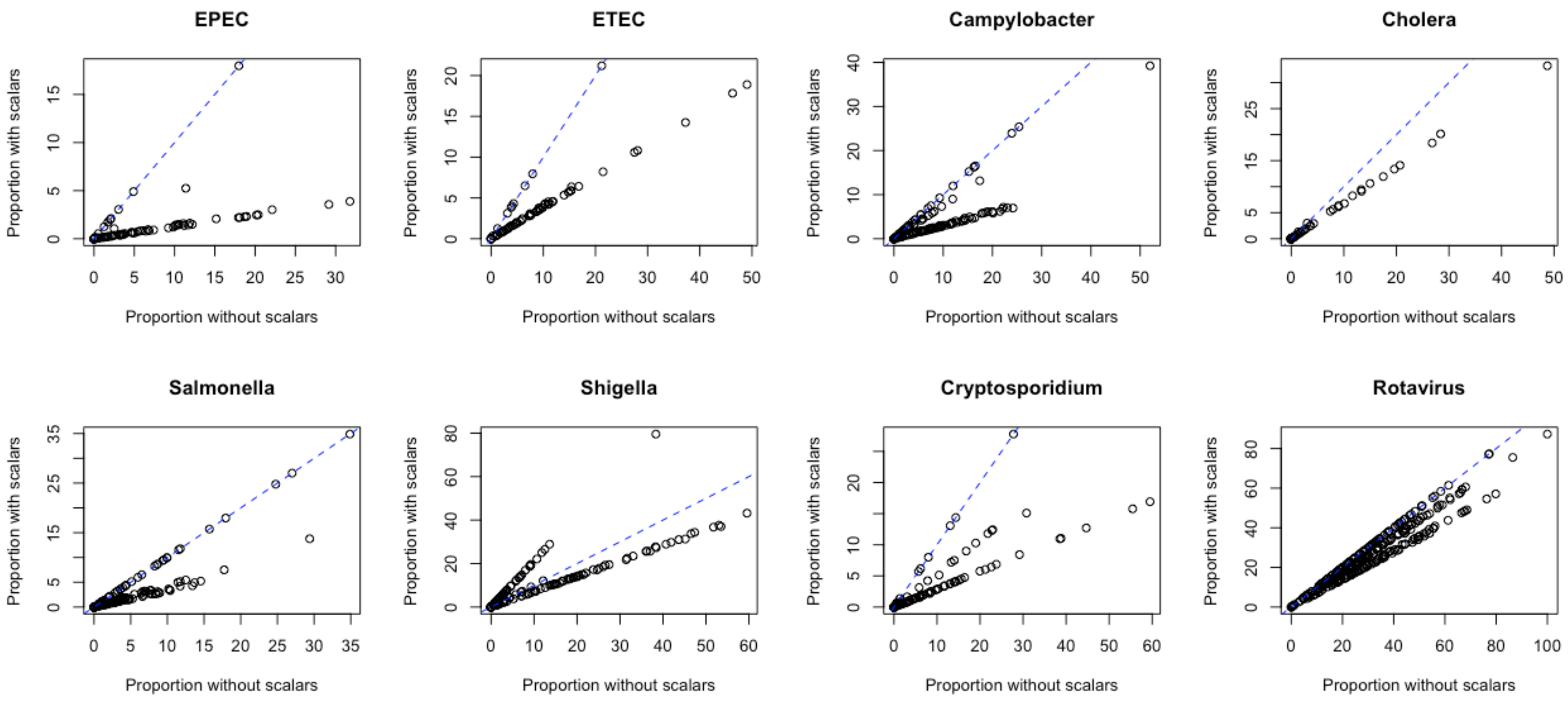

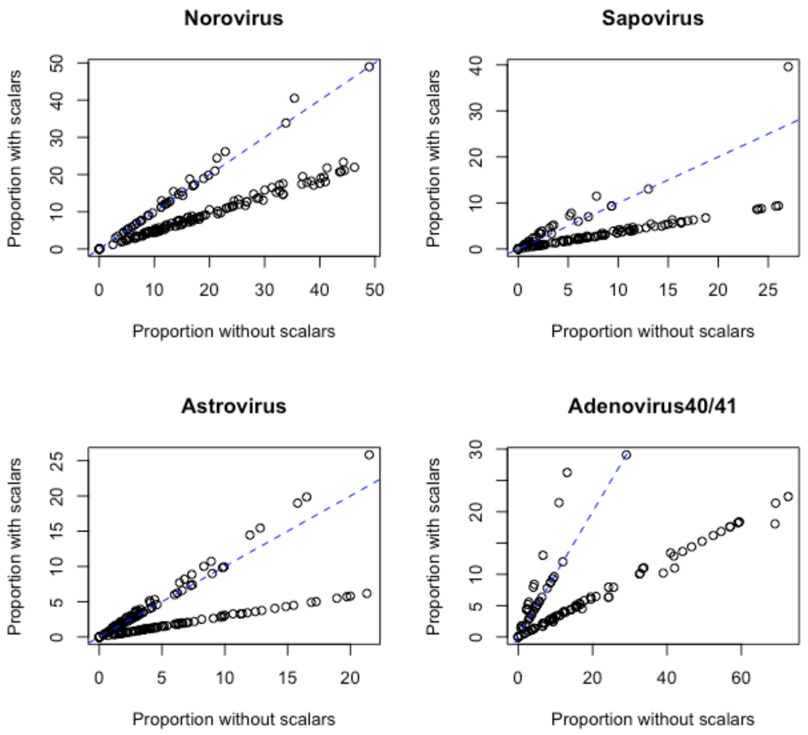

Norovirus
Sapovirus
Astrovirus
Adenovirus40/41
Proportion with scalars
Proportion without scalars

Web Appendix Statistical Methods. Details related to statistical methods

We examined additional covariates other than those included in our final Bayesian models, including year and under five mortality rate, with similar results (not shown). However, these additional covariates were not included due to high collinearity. Due to the large number of etiologies considered here, each additional covariate results in a considerable increase in the number of parameters to estimate. Unfortunately, some factors of interest were not reported in a systematic way among the literature identified in the systematic review, including diarrhea case definition, sampling method, and antibiotic usage. We did not examine indicators of healthcare access. The models included generally uninformative priors.

The coefficients in the Bayesian multinomial regression were estimated using the mean of the Markov Chain Monte Carlo estimates after a burn in period of 5000 iterations (for LMIC) and 10000 iterations (for HIC). For each country in each year, that country's region, rotavirus vaccine coverage, and GNI were used in a linear combination with these estimated coefficients for each pathogen, which was exponentiated for each pathogen and the residual "other" etiologies, scaled by the sum of these, to yield an estimated fraction of diarrhea deaths having values between zero and 1. This estimated fraction was applied to the estimated number of diarrhea deaths in each country year as previously reported, for the number of deaths for each pathogen in each country-year.

Overfitting in the Bayesian model was controlled using the Bayesian LASSO and the restriction parameter (lambda). We selected a moderate amount of restriction for both the high income and low-income models. We did not use cross validation to select the degree of restriction. In order to determine convergence of the Bayesian models, we examined the trace plots of estimated parameters, as well as the Gelman R statistic being less than 1.1. Trace plots are included below as Webappendix figures 4 and 5.

All input data and scripts related to statistical analysis are available at https://github.com/jamieperin/diarrhea_etiology.

Gelman A, Rubin DB. Inference from iterative simulation using multiple sequences. Statistical science. 1992 Nov;7(4):457-72.

Jags model definition:

```
model{
  for (s in 1:S){   # loop through studies

     ## Define model for each TRUE etiology
     # C number of TRUE etiology
     # q is log(odds) for each etiology: log(Pi/P1) where i = 1...C
```

```
    q[s,1] <- 1
    for(d in 2:C){
      log(q[s,d]) <- inprod(XM[s,1:K],B[1:K,d])
    }
    pi[s,1:C] <- q[s,1:C] / sum(q[s,1:C])

    P[fi[s]:la[s]] <- GM[fi[s]:la[s], 1:C] %*% pi[s,]

    ## Multinomial model of observed numbers in reported categories from expected proportions
    GM[fi[s]:la[s], C+1] ~ dmultinom(P[fi[s]:la[s]], N[s])
  }

  # Define priors
  for(c in 2:C){
      B[1,c] ~ dnorm(0,1/0.5^2)
   for(x in 1:K){
      B[x,c] ~ ddexp(0,lambda)
    }
  }
}
```

Web Appendix Figure 3. Model coefficient estimates

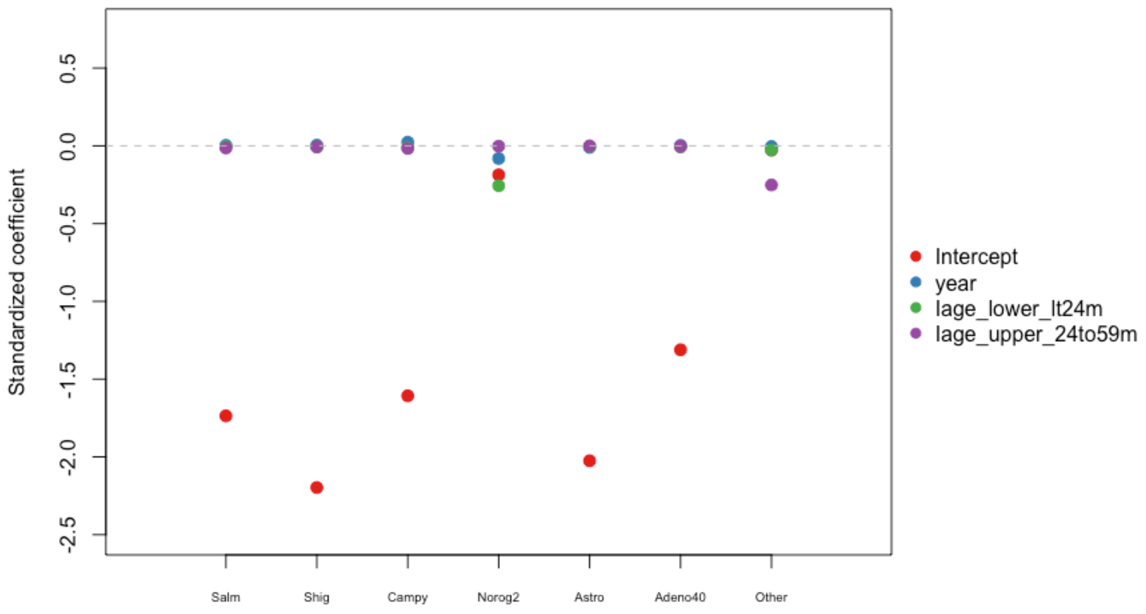

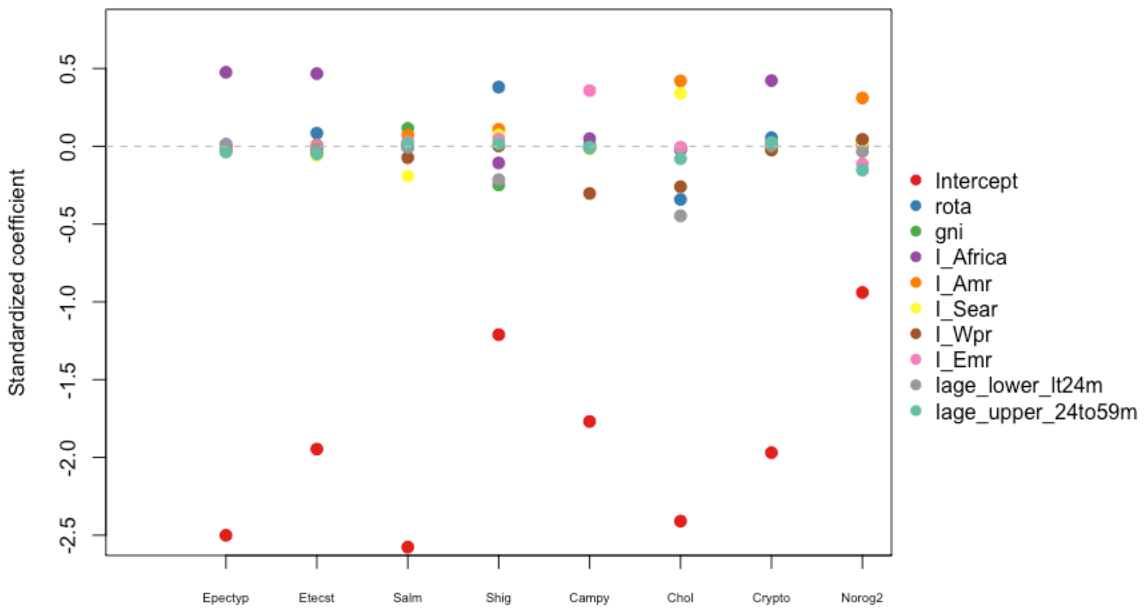

Standardized coefficient
0.5
0.0
-0.5
-1.0
-1.5
-2.0
-2.5
Epectyp
Etecst
Salm
Shig
Campy
Chol
Crypto
Norog2
Intercept
rota
gni
I_Africa
I_Amr
I_Sear
I_Wpr
I_Emr
lage_lower_lt24m
lage_upper_24to59m

Web Appendix Figure 4. Convergence for high income model.

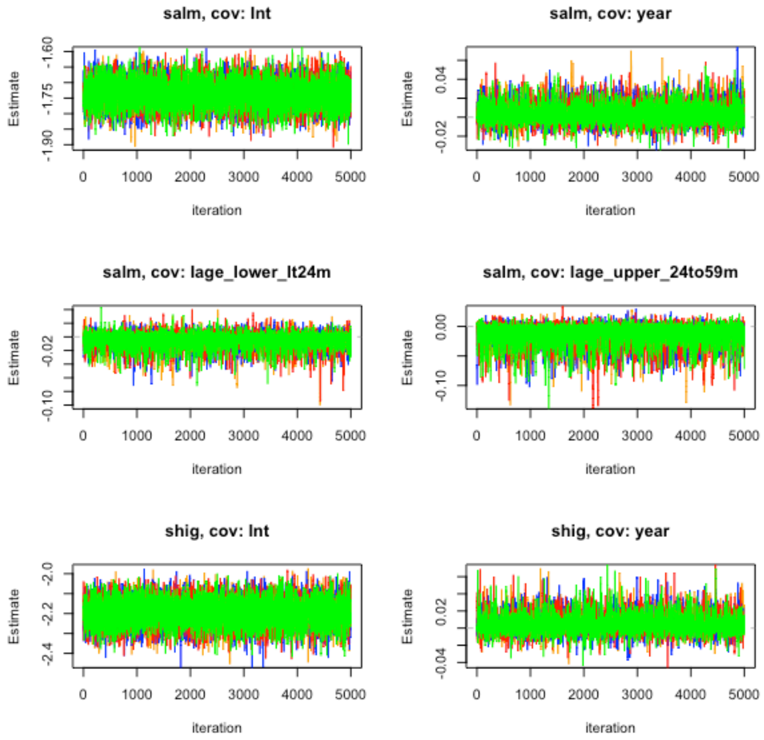

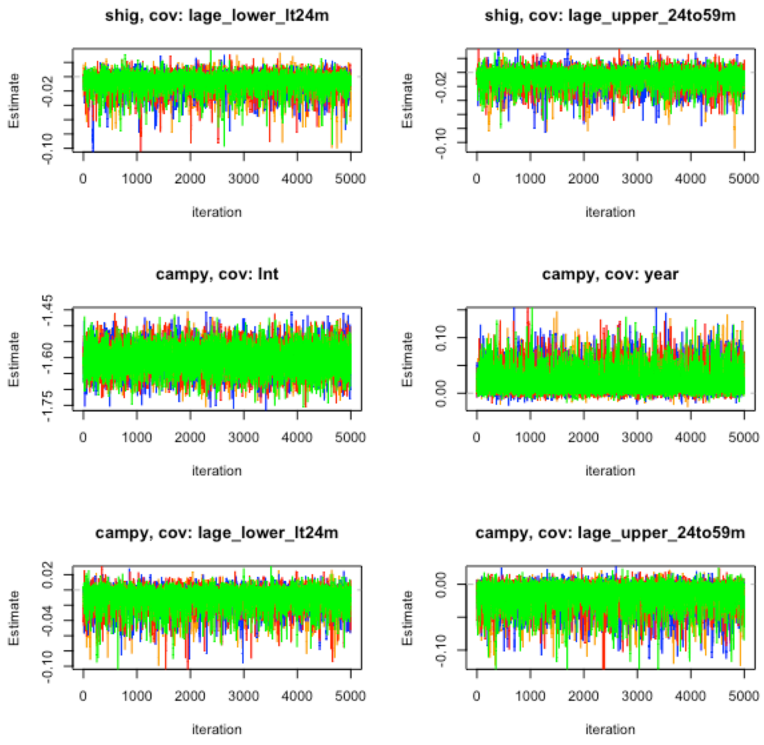
shig, cov: lage_lower_lt24m
shig, cov: lage_upper_24to59m
campy, cov: Int
campy, cov: year
campy, cov: lage_lower_lt24m
campy, cov: lage_upper_24to59m
Estimate
iteration
0
1000
2000
3000
4000
5000

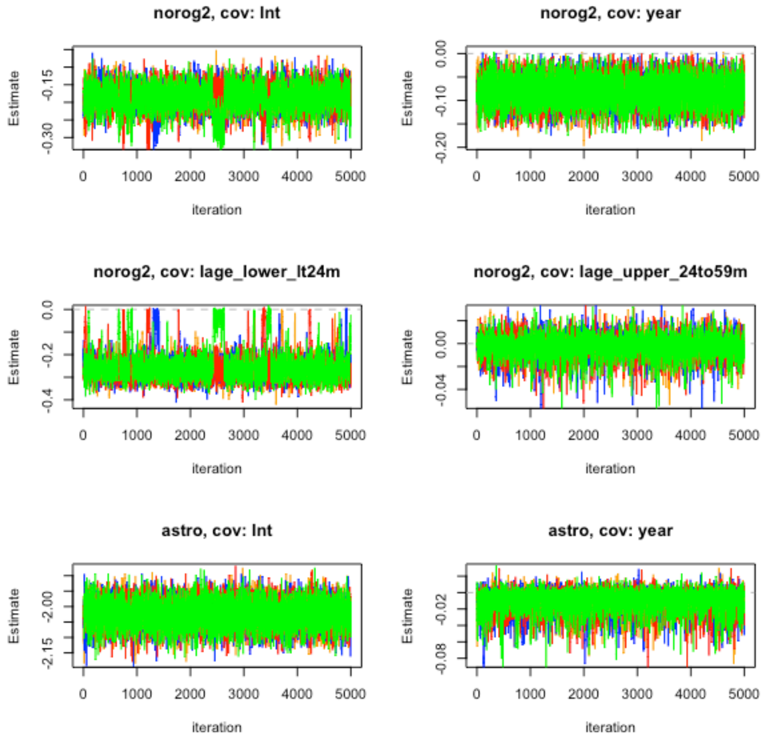
norog2, cov: Int
norog2, cov: year
norog2, cov: lage_lower_lt24m
norog2, cov: lage_upper_24to59m
astro, cov: Int
astro, cov: year
Estimate
iteration
0
1000
2000
3000
4000
5000

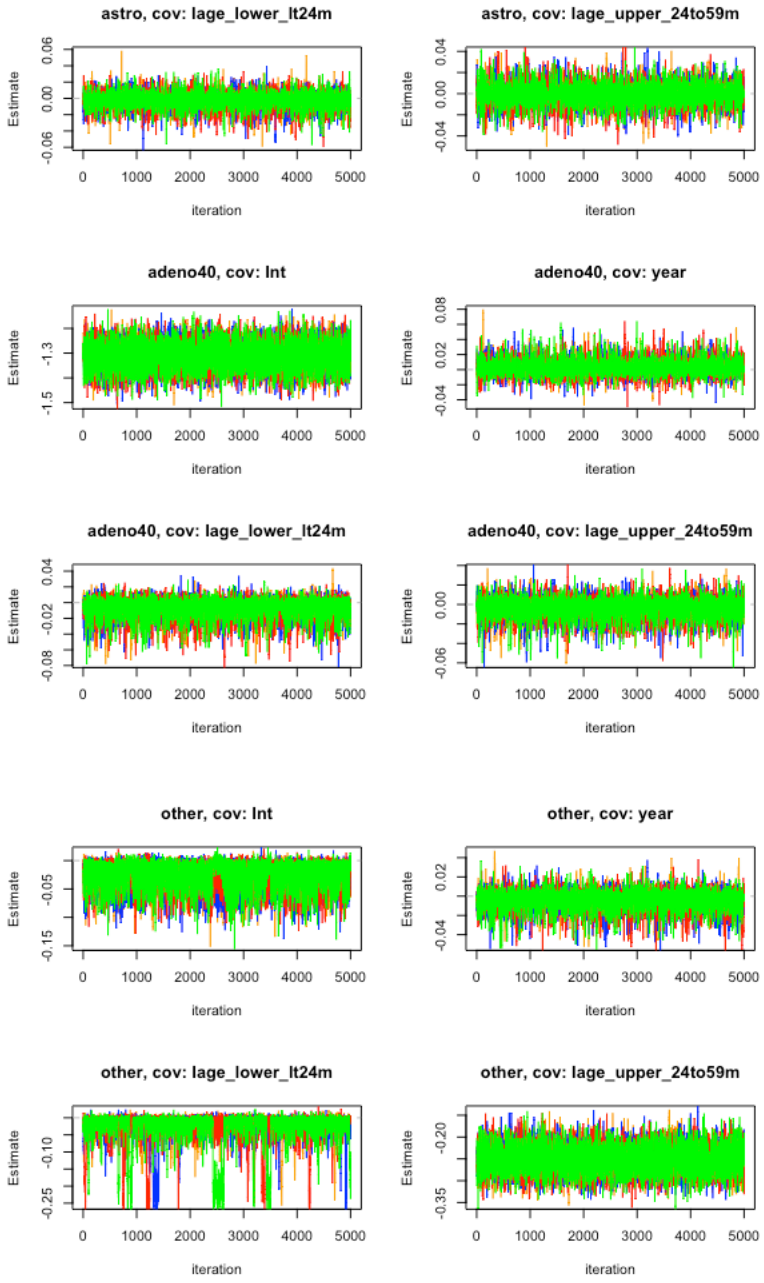
astro, cov: lage_lower_lt24m
astro, cov: lage_upper_24to59m
adeno40, cov: Int
adeno40, cov: year
adeno40, cov: lage_lower_lt24m
adeno40, cov: lage_upper_24to59m
other, cov: Int
other, cov: year
other, cov: lage_lower_lt24m
other, cov: lage_upper_24to59m
Estimate
iteration

Web Appendix Figure 5. Convergence for low and middle-income model.

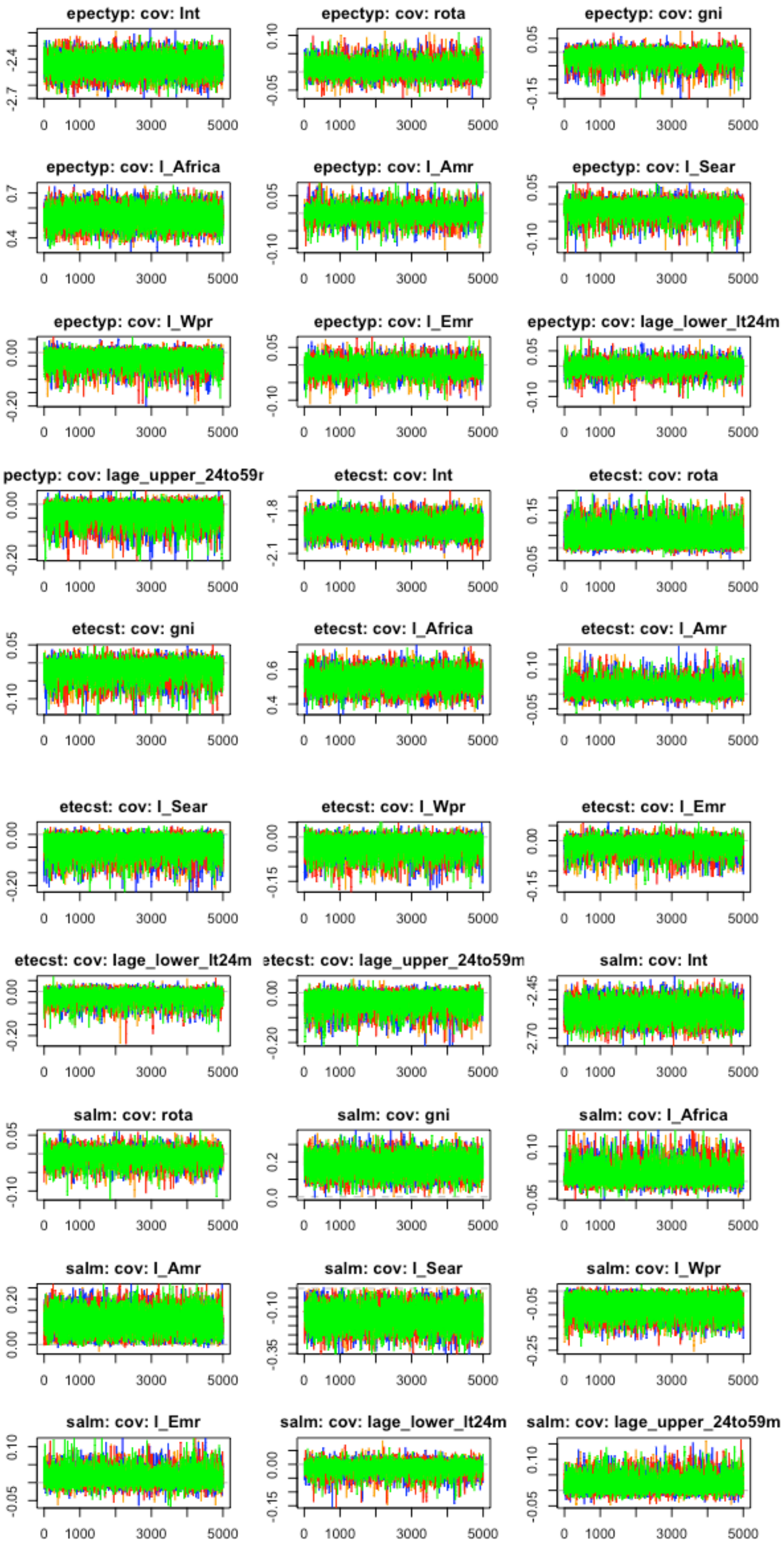

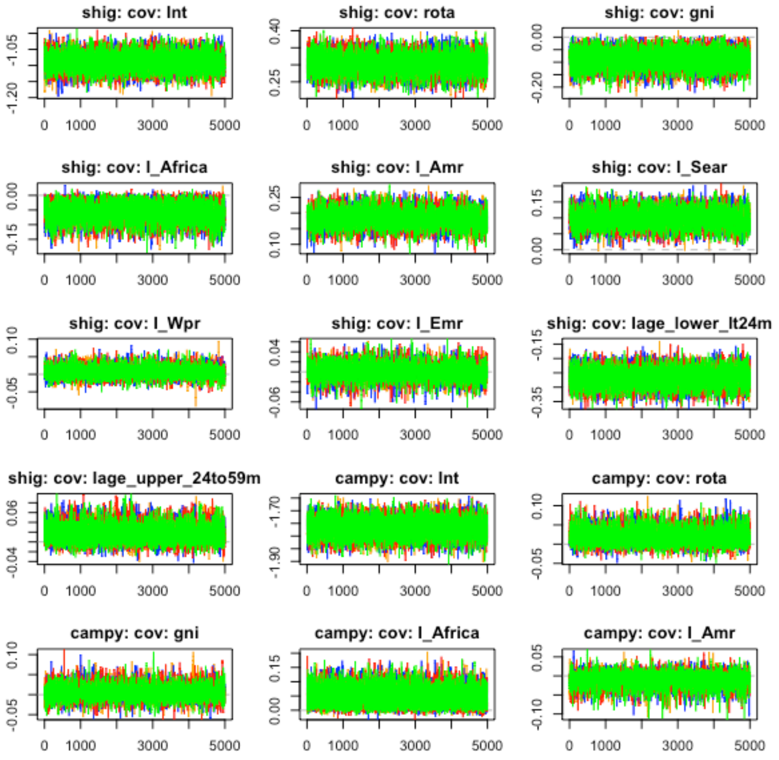
shig: cov: Int
shig: cov: rota
shig: cov: gni
shig: cov: l_Africa
shig: cov: l_Amr
shig: cov: l_Sear
shig: cov: l_Wpr
shig: cov: l_Emr
shig: cov: lage_lower_lt24m
shig: cov: lage_upper_24to59m
campy: cov: Int
campy: cov: rota
campy: cov: gni
campy: cov: l_Africa
campy: cov: l_Amr

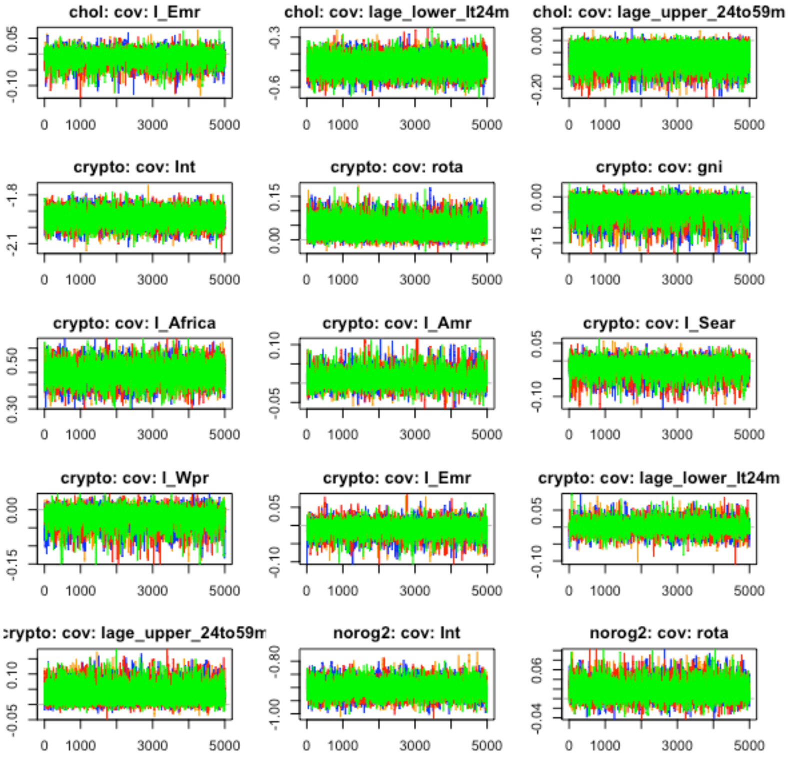
chol: cov: l_Emr
chol: cov: lage_lower_lt24m
chol: cov: lage_upper_24to59m
crypto: cov: Int
crypto: cov: rota
crypto: cov: gni
crypto: cov: l_Africa
crypto: cov: l_Amr
crypto: cov: l_Sear
crypto: cov: l_Wpr
crypto: cov: l_Emr
crypto: cov: lage_lower_lt24m
rypto: cov: lage_upper_24to59
norog2: cov: Int
norog2: cov: rota

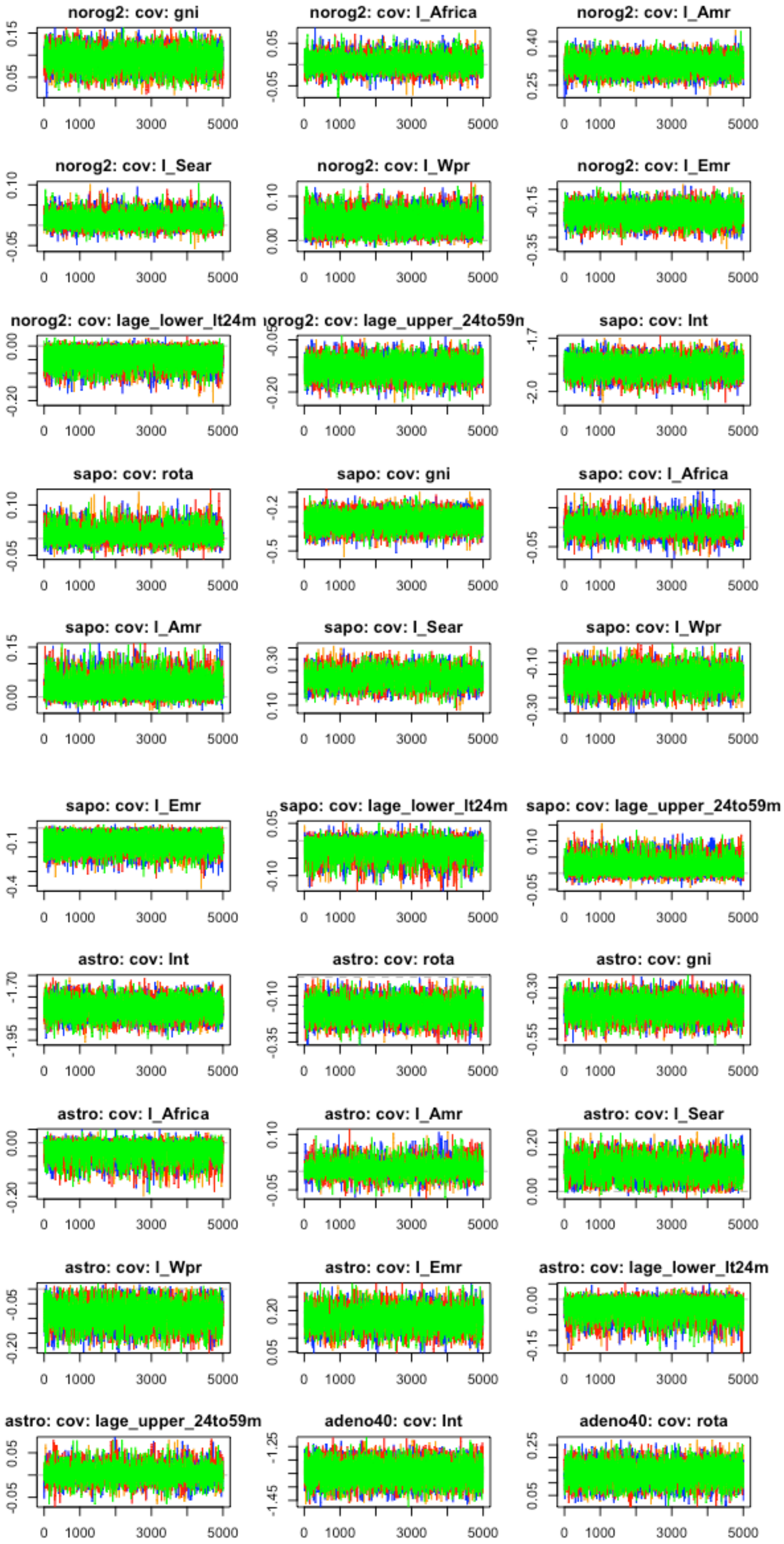
norog2: cov: gni
norog2: cov: l_Africa
norog2: cov: l_Amr
norog2: cov: l_Sear
norog2: cov: l_Wpr
norog2: cov: l_Emr
norog2: cov: lage_lower_lt24m
orog2: cov: lage_upper_24to59n
sapo: cov: Int
sapo: cov: rota
sapo: cov: gni
sapo: cov: l_Africa
sapo: cov: l_Amr
sapo: cov: l_Sear
sapo: cov: l_Wpr
sapo: cov: l_Emr
sapo: cov: lage_lower_lt24m
sapo: cov: lage_upper_24to59m
astro: cov: Int
astro: cov: rota
astro: cov: gni
astro: cov: l_Africa
astro: cov: l_Amr
astro: cov: l_Sear
astro: cov: l_Wpr
astro: cov: l_Emr
astro: cov: lage_lower_lt24m
astro: cov: lage_upper_24to59m
adeno40: cov: Int
adeno40: cov: rota

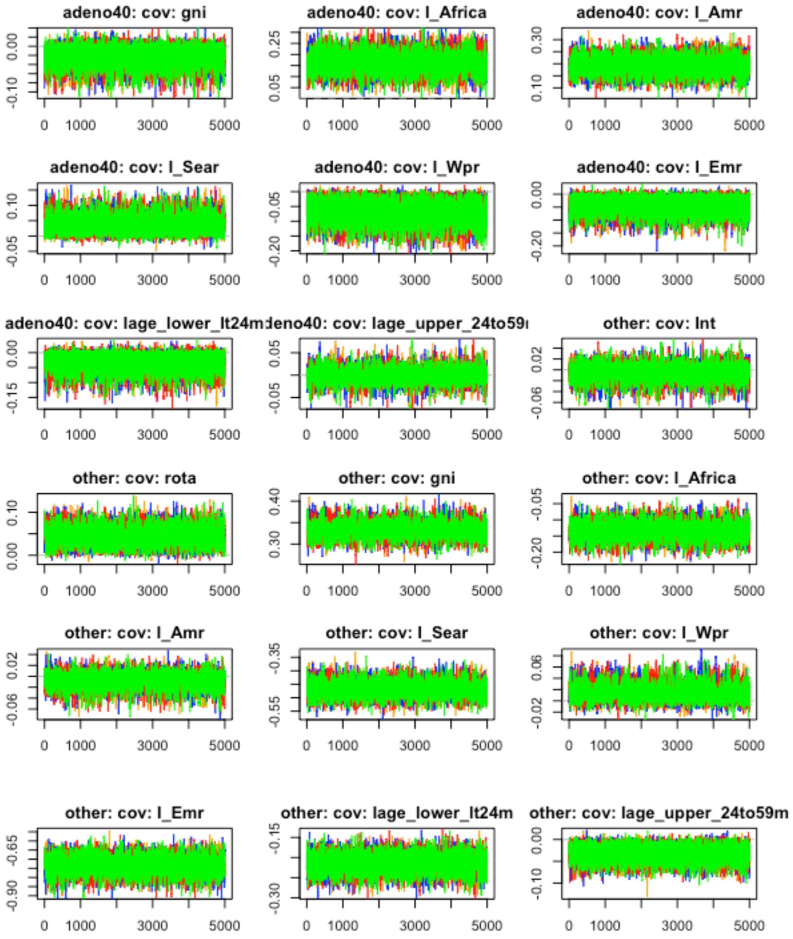
adeno40: cov: gni
adeno40: cov: l_Africa
adeno40: cov: l_Amr
adeno40: cov: l_Sear
adeno40: cov: l_Wpr
adeno40: cov: l_Emr
adeno40: cov: lage_lower_lt24m
deno40: cov: lage_upper_24to59
other: cov: Int
other: cov: rota
other: cov: gni
other: cov: l_Africa
other: cov: l_Amr
other: cov: l_Sear
other: cov: l_Wpr
other: cov: l_Emr
other: cov: lage_lower_lt24m
other: cov: lage_upper_24to59m

Web Appendix Table 4. Comparison of Previous[6,11,25] and current estimates of the etiologies of diarrhea deaths

Cause	2011*<br>Lanata[6]<br>(%)	2017-18 †<br>Cohen[11]<br>(%)	2019‡<br>GBD[25]<br>(%)	2021§<br>Black<br>(%)
Rotavirus	27·8	35·7	30·3	24·4
Shigella sp	3·9	10·8	18·7	8·1
Norovirus	9·9	6·2¶	-	7·4¶
Adenovirus 40/41	3·1	3·9	16·7	7·4
ETEC	6·0	3·9¶	2·5	6·4¶
Cryptosporidium sp	2·0	3·4	15·4	5·8
Campylobacter jejuni/coli	3·2	1·7	11·8	5·5
Astrovirus	2·1	3·0	-	4·8
Sapovirus	-	3·9¶	-	4·7¶
EPEC	11·1	-	3·2	3·7
Salmonella sp	2·5	1·0	7·9	1·8
Vibrio cholerae	1·3	-	11·3	1·4
Other	24·5	27·4	-	18·7

*Year for which estimates done
†Total of estimated causes exceeds 2017-18 total diarrhea deaths by 3·3%
‡Total of estimated causes exceeds 2019 total diarrhea deaths by 26%
§Total of estimated causes exceeds 2021 total diarrhea deaths by 0·1% due to rounding
¶Restricted to norovirus GII, ST-ETEC and typical EPEC
